# Oxidative stress antagonizes fluoroquinolone drug sensitivity via the SoxR-SUF Fe-S cluster homeostatic axis

**DOI:** 10.1371/journal.pgen.1009198

**Published:** 2020-11-02

**Authors:** Audrey Gerstel, Jordi Zamarreño Beas, Yohann Duverger, Emmanuelle Bouveret, Frédéric Barras, Béatrice Py

**Affiliations:** 1 Laboratoire de Chimie Bactérienne, Aix-Marseille Université-CNRS UMR7283, Institut de Microbiologie de la Méditerranée, Marseille, France; 2 SAMe Unit, Département de Microbiologie, Institut Pasteur, CNRS UMR IMM 2001, Paris, France; Swiss Federal Institute of Technology Lausanne (EPFL), SWITZERLAND

## Abstract

The level of antibiotic resistance exhibited by bacteria can vary as a function of environmental conditions. Here, we report that phenazine-methosulfate (PMS), a redox-cycling compound (RCC) enhances resistance to fluoroquinolone (FQ) norfloxacin. Genetic analysis showed that *E*. *coli* adapts to PMS stress by making Fe-S clusters with the SUF machinery instead of the ISC one. Based upon phenotypic analysis of *soxR*, *acrA*, and *micF* mutants, we showed that PMS antagonizes fluoroquinolone toxicity by SoxR-mediated up-regulation of the AcrAB drug efflux pump. Subsequently, we showed that despite the fact that SoxR could receive its cluster from either ISC or SUF, only SUF is able to sustain efficient SoxR maturation under exposure to prolonged PMS period or high PMS concentrations. This study furthers the idea that Fe-S cluster homeostasis acts as a sensor of environmental conditions, and because its broad influence on cell metabolism, modifies the antibiotic resistance profile of *E*. *coli*.

## Author summary

Our study investigates how phenazine compounds, which are widely present in the environment, impact antibiotic resistance of the Gram-negative bacteria *Escherichia coli*. The paucity of new antibacterial molecules fuels concern in the wake of increased antibiotic resistance among pathogens. Equally worrying is the realization that environmental conditions can have a drastic influence on the efficiency of antibacterial compounds. Here we report that phenazine, a member of the redox-cycling molecule family, is antagonistic to norfloxacin, a well-known and routinely used fluoroquinolone antibiotic. We show that the mechanism *E*. *coli* is using for synthesizing Fe-S clusters controls the phenazine/fluoroquinolone antagonism. Indeed, upon exposure to phenazine, *E*. *coli* switches from making Fe-S clusters with the ISC Fe-S biogenesis system to making them with SUF, a consequence of which is the activation of the SoxR transcriptional activator, up-regulation of the AcrAB efflux pump, and efflux of fluoroquinolone out of the cell. This study illustrates the major influence that environmental conditions play in setting antibiotic level resistance and further highlights the major contribution of Fe-S cluster homeostasis in antibiotic susceptibility.

## Introduction

Drug combination is a potent strategy against the worrying rise of multi-drug resistant bacteria as it reduces the chance of resistance acquisition [1,2]. However, several instances of antagonisms between drugs have been reported [3,4]. In fact, a thorough investigation of growth phenotypes caused by pair-wise combination of over 250 compounds, including neglected antibiotics, FDA approved human drugs and food additives, revealed antagonism to be more prevalent than synergy [5]. For instance, drugs causing oxidative stress, such as paraquat or plumbagin, were found to antagonize antibiotics of different families including quinolones [5]. This was consistent with the previous observation that redox-cycling compounds (RCC) such as paraquat or plumbagin enhanced both survival and persister formation in the presence of the oxolinic acid fluoroquinolone [6,7].

Previously, we reported antagonism between iron scavengers such as 2,2’ dipyridyl (DIP) and aminoglycosides toxicity in *Escherichia coli* [8]. At the molecular level, DIP/aminoglycoside antagonism was shown to be orchestrated by Fe-S cluster-homeostasis regulation. Briefly, Fe-S clusters rank among the most conserved prosthetic groups that rely on dedicated machineries to be built and transferred to client proteins. *E*. *coli* possesses two such machineries, ISC and SUF, which synthesize and deliver Fe-S clusters to about 150 apo-proteins. ISC and SUF machineries function following the same basic principles. Cysteine desulfurases (IscS, SufS) produce sulfur from L-cysteine, scaffold proteins (IscU, SufBC_2_D) provide a molecular platform allowing iron and sulfur to meet and form a cluster, and carrier proteins—such as, IscA, SufA, ErpA and NfuA—deliver the cluster to terminal apotargets [9–14]. The source of iron remains uncertain and multiple origins have been proposed such as frataxin [9]. ISC is the housekeeping machinery, employed during balanced growth conditions, and SUF is the stress-responding one [9–14]. Mutants lacking both ISC and SUF are not viable [15]. Under iron limitation, *E*. *coli* makes clusters with SUF and those SUF-using cells exhibit enhanced phenotypic resistance to aminoglycoside. Indeed, SUF is inefficient at targeting clusters to the proton-motive force (pmf)-producing respiratory complexes, and a reduced aminoglycoside uptake ensues [8].

Following our analysis of the role played by Fe-S cluster homeostasis in DIP/aminoglycoside antagonism, we decided to investigate the role of Fe-S cluster-homeostasis within the RCC/fluoroquinolones antagonism, in particular the role of SoxR. Indeed, the SoxR transcriptional factor, which uses Fe-S cluster to sense redox changes, was suggested to intervene in the antagonism between oxidative stress and quinolone resistance or tolerance [6,7,16–18].

This led us to show that strains exposed to RCC make clusters with the SUF system, which matures and permits activation of SoxR transcriptional activator under oxidative stress. In turn, oxidized SoxR up-regulates the AcrAB efflux pump that likely expels fluoroquinolone. Hence, these results and our previous study show that exposure to toxic chemicals in the environment, such as RCC or DIP, both cause a switch from ISC to SUF, yielding enhanced resistance to antibiotics. However, while the DIP/aminoglycoside antagonism resulted from reduced uptake of aminoglycoside, the RCC/fluoroquinolone antagonism comes from enhanced export of fluoroquinolone.

## Results

### The SoxR regulon confers PMS-mediated protection against quinolones

**Fig 1:**
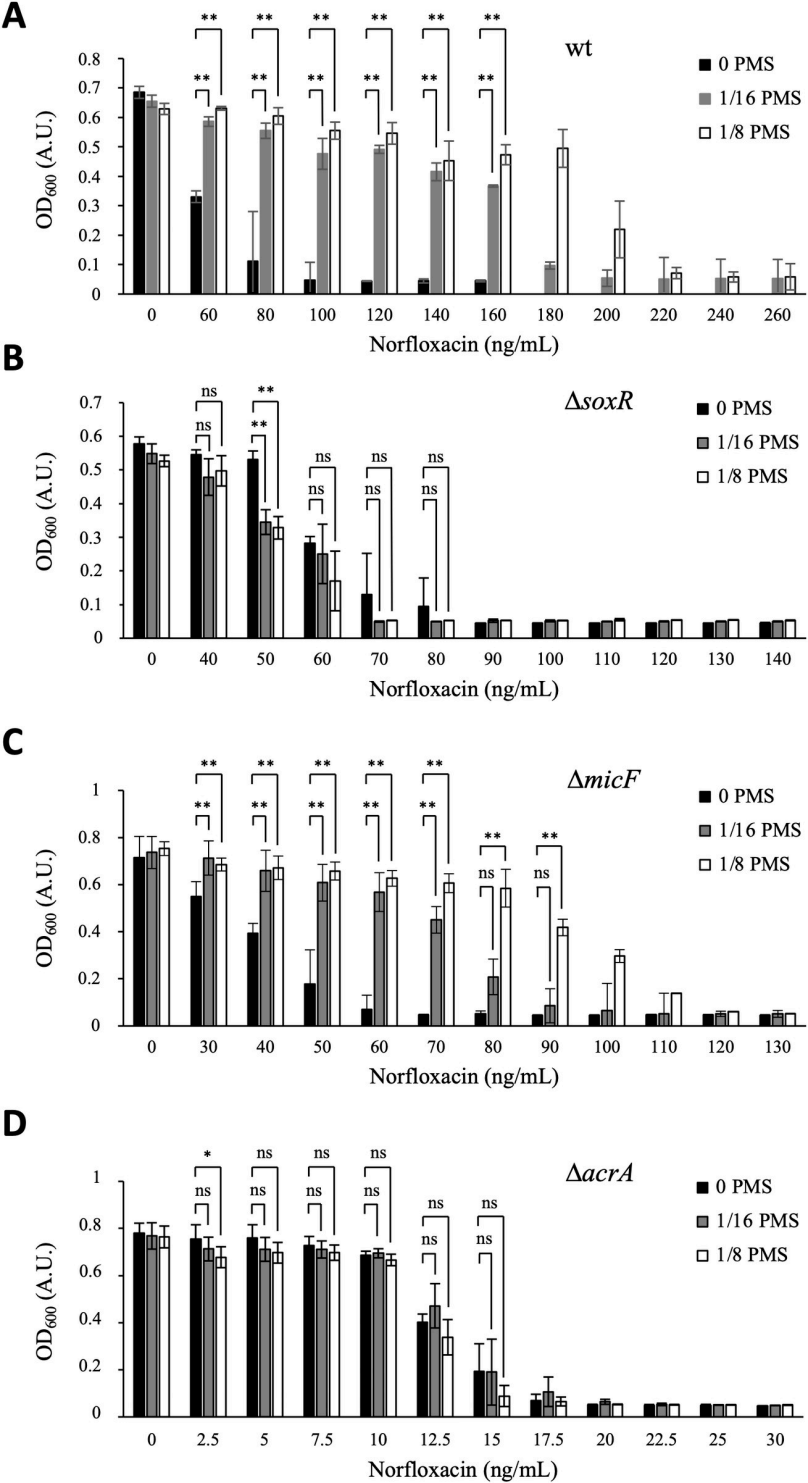
PMS-mediated protection against norfloxacin is dependent on SoxR. The *E*. *coli* wild type (wt) (BE1000) (A), Δ*soxR* (AG035) (B), Δ*micF* (YD002) (C) and Δ*acrA* (YD001) (D) strains were grown to mid-log phase in LB and then diluted to inoculate 96-well microplate wells containing LB liquid medium supplemented with norfloxacin at the indicated final concentration and supplemented or not (black bars) with PMS at 1/16 (grey bars) and 1/8 (white bars) of the MIC_PMS_ of each strain (MIC_PMS_ were 140 μM for wt, 40 μM for Δ*soxR*, 100 μM for Δ*micF* and 15 μM for Δ*acrA*). Cultures were incubated 18 hours at 37°C with shaking. Plates were read for OD_600_ in Tecan Infinite. The experiment was repeated at least three times. The means and standard deviations are shown. Asterisks represent the statistical significance calculated using the Bonferroni method (** p<0.01; * p<0.05; ns p>0.05).

To investigate the molecular basis of the oxidative stress/quinolone antagonism, we choose phenazine-methosulfate (PMS) as RCC, and norfloxacin as fluoroquinolone. To test whether SoxR intervenes in the PMS/norfloxacin antagonism, wt and Δ*soxR* strains were exposed to norfloxacin, alone or in combination with sub-inhibitory concentrations of PMS (1/8 and 1/16 of the MIC_PMS_ of each strain; MIC_PMS_ were 140 μM for wt; 40 μM for Δ*soxR*). Adding PMS at a concentration of 1/8 MIC_PMS_ led to a drastic enhancement of norfloxacin resistance level of the wt strain (Fig 1A). Indeed, when treated with norfloxacin concentration between 100 and 180 ng/mL, the wt strain exposed to PMS (concentration at 1/8 MIC_PMS_ value) reached OD_600_ values between 0.4–0.6, while in the absence of PMS, OD_600_ values barely reached 0.1 (Fig 1A). Even at 1/16 MIC_PMS_ concentration, PMS remained a potent antagonist of norfloxacin (Fig 1A). In sharp contrast, PMS exerted no antagonistic effect on norfloxacin in the Δ*soxR* mutant (Fig 1B). SoxR activates the expression of the *soxS* gene, which in turn activates target genes involved in antibiotic resistance and superoxide resistance [16–26]. Interestingly, we showed that expression of an IPTG-inducible allele of *soxS* on a plasmid enhanced fluoroquinolone resistance levels of both wt and Δ*soxR* mutant (Table 1), mimicking the PMS antagonistic effect, further strengthening the conclusion that the PMS/norfloxacin antagonism involves SoxS-activated genes.

**Table 1:** SoxS-mediated resistance against norfloxacin.

	MIC_Norfloxacin_ (ng/mL) (The means and standard deviations are indicated)	
	pTrc99A	pSoxS
wt (BE1000)	127.5 (+/- 9.6)	825 (+/- 28.9)
Δ*soxR* (AG035)	100 (+/- 0)	875 (+/- 28.9)

The effect of the PMS/norfloxacin drug combination was analyzed in mutants of two targets of the SoxRS regulatory system, *micF* and *acrA*. The *micF* gene encodes a small RNA that negatively regulates translation of the OmpF porin, hence its induction could potentially prevent OmpF-mediated entry of PMS [27]. However, PMS-mediated drug antagonism was still observed in the Δ*micF* mutant (Fig 1C). The *acrA* gene encodes a major multidrug efflux pump (AcrAB-TolC) that includes fluoroquinolones (FQ) as a substrate [28,29]. The PMS antagonistic effect on FQ action was completely lost in the Δ*acrA* mutant (Fig 1D). By qRT-PCR, we found that expression of the *acrA* and *acrB* genes was up-regulated (2- to 3-fold) in cells treated with either PMS only, or with both PMS and norfloxacin (Table 2). In contrast, treatment with norfloxacin only exerted, if anything, a down-regulation of 1.4-fold of *acrA* and *acrB* gene expression (Table 2). Altogether these results support the view that PMS protects *E*. *coli* from norfloxacin, by activating AcrAB-mediated efflux of the fluoroquinolone *via* the SoxRS regulatory system.

**Table 2:** qRT-PCR analysis of the expression of *acrA* and *acrB* genes.

	Fold change expression treated/untreated	
	*acrA*	*acrB*
Norfloxacin	0.73 (+/- 0.03)	0.7 (+/- 0.03)
PMS	2.99 (+/- 0.25)	2.7 (+/- 0.29)
PMS + Norfloxacin	2.42 (+/- 0.17)	2.09 (+/- 0.13)

### The SUF machinery is required to sustain growth in the presence of PMS

**Fig 2:**
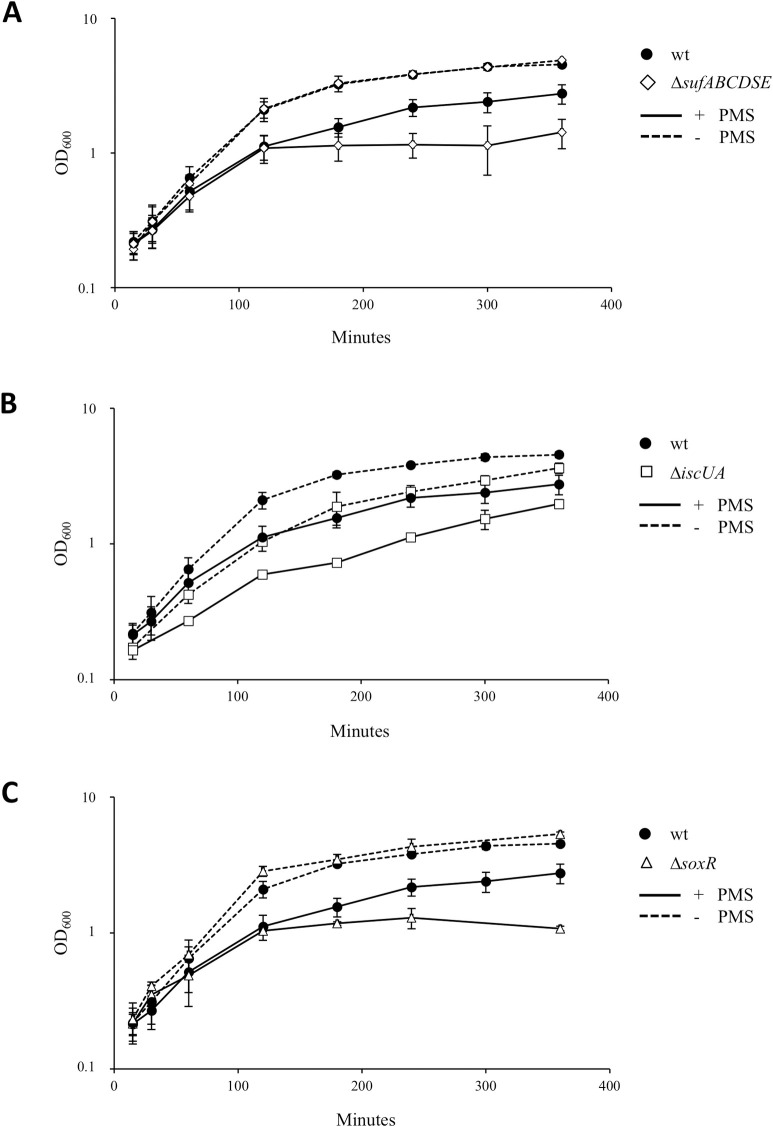
The Δ*sufABCDSE* mutant is hypersensitive to PMS. The *E*. *coli* wt (BE1000) (black circles) (A-C), Δ*sufABCDSE* (AG031) (white diamonds) (A), Δ*iscUA* (AG030) (white squares) (B) and Δ*soxR* (AG035) (white triangles) (C) strains were grown overnight in LB and inoculated (1/100) in fresh LB medium. The cultures were grown to an OD_600_ of 0.2 and were each split into two flasks, PMS (30 μM) was added (time zero) in one, and the other was left untreated. All cultures were further incubated at 37°C and growth was monitored by following OD_600_. The growth curves of the wt strain are the same in each panel. The experiments were repeated at least three times. The means and standard deviations are shown.

SoxR being an Fe-S cluster-containing protein, we went further in analyzing the importance of ISC and SUF Fe-S clusters biogenesis systems in PMS stress and in the PMS/norfloxacin antagonism. Previous work has reported that the *iscRSUA* and *sufABCDSE* operons are induced by PMS [30,31], which we confirmed here by using P*iscR*::*lacZ* and P*sufA*::*lacZ* gene fusions (2.7 and 3.3-fold increase in the presence of 20 μM PMS, respectively) (S1 Fig). Then, the effect of PMS on the growth kinetics of LB grown cultures was tested. PMS (30 μM) was added during the exponential growth phase and OD_600_ values were recorded (Fig 2). Two hours after addition of PMS, we observed that the Δ*sufABCDSE* and the Δ*soxR* mutants stopped growing whereas growth of the wt strain did not (Fig 2A and 2C). In the presence of PMS, growth of the Δ*iscUA* mutant appeared to be slower than the wt strain but this is a general feature of the Δ*iscUA* mutant even under non-stressed conditions (Fig 2B) (in untreated condition, the doubling times of the Δ*iscUA* mutant and the wt strain were 35 and 28 min, respectively), and more importantly, like the wt, it did not cease growing (Fig 2B).

RCC such as PMS are predicted to enhance intracellular level of superoxide [32]. An enhanced level of intracellular superoxide stress can be obtained by using strains lacking superoxide dismutase SodA or/and SodB. As expected, the Δ*sodA*, Δ*sodA* Δ*sodB* mutants and to some extent the single Δ*sodB* mutant showed hypersensitivity to PMS (S2 Fig). Introduction of the Δ*sufABCDSE* mutation in Δ*sodA*, Δ*sodB*, or Δ*sodA* Δ*sodB* mutants increased drastically the sensitivity to PMS (S2 Fig). In contrast, combining Δ*iscUA* mutation with Δ*sodA*, Δ*sodB* or both Δ*sodA* Δ*sodB* mutations did not enhance PMS sensitivity (S2 Fig). Altogether, these results established the importance of the SUF system in allowing *E*. *coli* to resist PMS stress and by inference, revealed that ISC was of no help in these conditions.

### Impact of the SUF and ISC machineries on the PMS-mediated protection from norfloxacin

**Fig 3:**
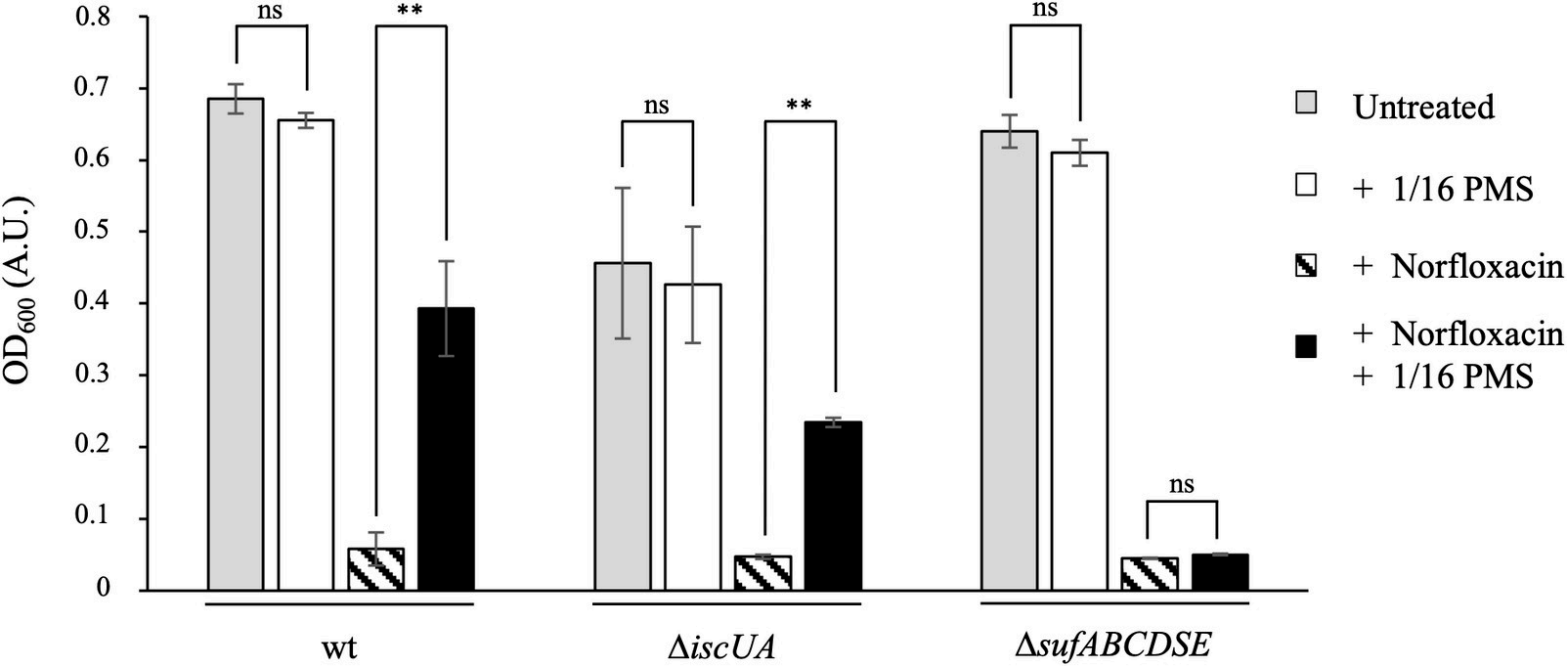
PMS-mediated protection against norfloxacin is altered in the Δ*sufABCDSE* mutant. The *E*. *coli* wt (BE1000), Δ*iscUA* (AG030) and Δ*sufABCDSE* (AG031) strains were grown to mid-log phase in LB and then diluted to inoculate 96-well microplate wells containing LB liquid medium (grey bars), LB medium supplemented with 1/16 of the MIC_PMS_ of each strain (white bars) (MIC_PMS_ were 140 μM for wt, 160 μM for Δ*iscUA* and 55 μM for Δ*sufABCDSE*), LB medium supplemented with norfloxacin (150 ng/mL) (hatched bars) and LB medium supplemented with both 1/16 of the MIC_PMS_ of each strain and norfloxacin (150 ng/mL) (black bars). Cultures were incubated 18 hours at 37°C with shaking. Plates were read for OD_600_ in Tecan Infinite. The experiments were repeated at least three times. The means and standard deviations are shown. Asterisks represent the statistical significance calculated using the Bonferroni method (** p<0.01; * p<0.05; ns p>0.05).

**Fig 4:**
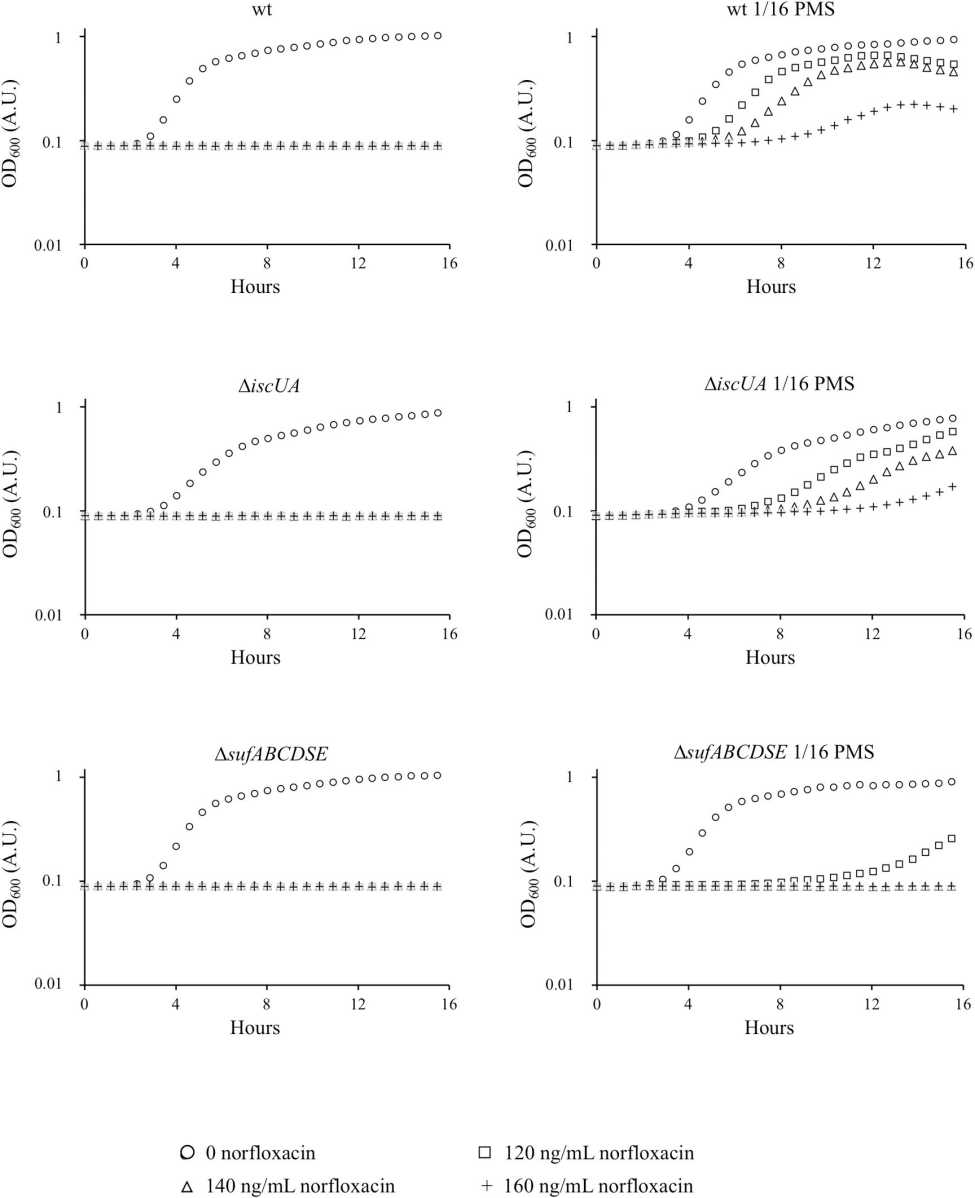
Effect of PMS on the growth of norfloxacin treated cells. The *E*. *coli* wt (BE1000) (A), Δ*iscUA* (AG030) (B) and Δ*sufABCDSE* (AG031) (C) strains were grown to mid-log phase in LB and then diluted to inoculate fresh LB medium (1 x 10^5^ c.f.u./mL), in 96-well microplate, supplemented or not (circles) with norfloxacin (final concentrations; 120 ng/mL squares; 140 ng/mL triangles; 160 ng/mL crosses) in the presence (right panels) or not (left panels) of PMS (1/16 of the MIC_PMS_ of each strains; MIC_PMS_ were 140 μM for wt; 160 μM for Δ*iscUA*; 55 μM for Δ*sufABCDSE*). Cultures were incubated at 37°C with shaking. Plates were read for OD_600_ in Tecan Infinite. The experiments were repeated at least three times. Complete growth curves of a representative experiment are shown. Corresponding growth parameters are indicated in Table 3.

Contribution of ISC and SUF in PMS/norfloxacin antagonism was then investigated. First, we showed that the PMS-induced resistance to norfloxacin occurred in the wt strain and the Δ*iscUA* mutant, whereas it was not observed in the Δ*sufABCDSE* mutant (Fig 3). Sub-inhibitory concentration of PMS (1/16 MIC_PMS_ concentration) permitted both the wt and Δ*iscUA* strains to grow in the presence of increasing concentrations of norfloxacin (120 ng/mL, 140 ng/mL, 160 ng/mL) (Fig 4 and Table 3). In contrast, sub-inhibitory concentration of PMS did not protect the Δ*sufABCDSE* mutant above 120 ng/mL norfloxacin (Fig 4). All growth parameters (lag phase, growth rate, final OD_600_) were more severely affected in the Δ*sufABCDSE* mutant than in the wt and Δ*iscUA* strains (Fig 4 and Table 3), illustrating that the protection against norfloxacin afforded by PMS depends upon a functional SUF system.

**Table 3:** Effect of PMS on the growth rate of norfloxacin treated cells.

		No PMS added	1/16 PMS
	Norfloxacin (ng/mL)	Growth rate (h^-1^)	Growth rate (h^-1^)
wt (BE1000)	0	0.75 (+/- 0.03)	0.66 (+/- 0.02)
	120	0	0.51 (+/- 0.01)
	140	0	0.47 (+/- 0.07)
	160	0	0.34 (+/- 0.13)
Δ*iscUA* (AG030)	0	0.46 (+/- 0.03)	0.35 (+/- 0.01)
	120	0	0.28 (+/- 0.01)
	140	0	0.28 (+/- 0.03)
	160	0	0.27 (+/- 0.03)
Δ*sufABCDSE* (AG031)	0	0.75 (+/- 0.03)	0.71 (+/- 0.03)
	120	0	0.2 (+/- 0.1)
	140	0	0
	160	0	0

### SUF, but not ISC, is required for SoxR maturation under PMS stress

**Fig 5:**
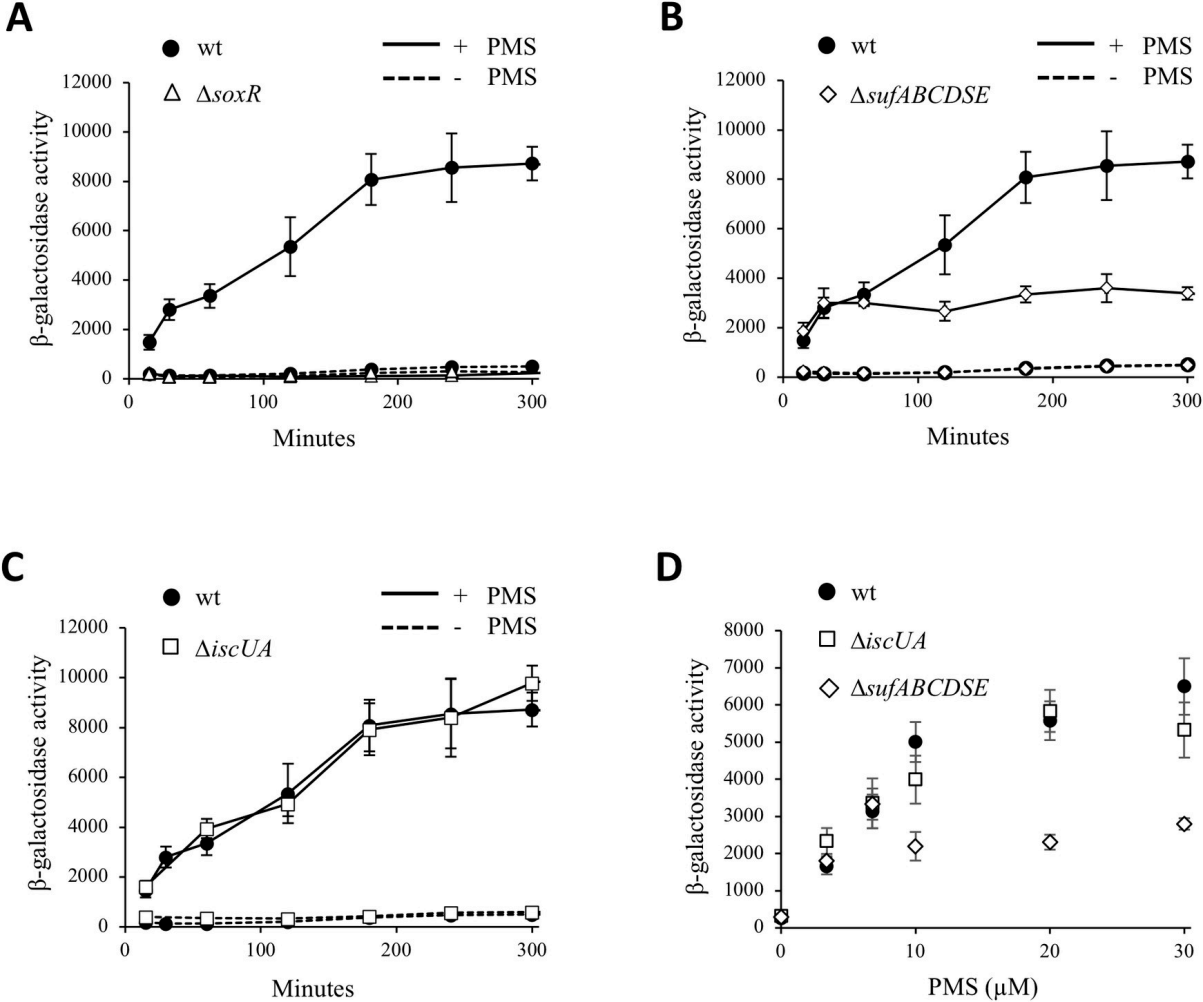
Maturation of SoxR requires the SUF machinery during PMS stress. Expression of the chromosomal P*soxS*::*lacZ* fusion was analyzed in *E*. *coli* wt (BE1000) (black circles) (A-C), Δ*soxR* (AG035) (white triangles) (A), Δ*sufABCDSE* (AG031) (white diamonds) (B) Δ*iscUA* (AG030) (white squares) (C) strains. Bacteria were grown overnight in LB and inoculated (1/100) in fresh LB medium. The cultures were grown to an OD_600_ of 0.2, and were each split into two flasks, PMS (30 μM) was added (time zero) in one, and the other was left untreated. All cultures were further incubated at 37°C with shaking and β-galactosidase activity was monitored and expressed as Miller units. (D) The *E*. *coli* strains carrying the chromosomal P*soxS*::*lacZ* fusion, wt (BE1000) (black circles), Δ*iscUA* (AG069) (white squares) and AG031 (Δ*sufABCDSE*) (white diamonds) were grown overnight in LB and inoculated (1/100) in fresh LB medium. The cultures were grown to an OD_600_ of 0.2, and PMS (3.4; 6.8; 10; 20 and 30 μM) was added or not. All cultures were further incubated for 2 hours at 37°C with shaking and β-galactosidase activity was measured and expressed as Miller units. The experiments were repeated at least three times. The means and standard deviations are shown.

Results above showed that both SUF and SoxR were required for the PMS/norfloxacin antagonism. A hypothesis was that PMS generates conditions during which SUF is required for synthesizing and carrying Fe-S clusters to SoxR, hence permitting its transcriptional activator function to fire anti-fluoroquinolone defense genes, including the AcrA pump. To test which system, ISC or SUF, was used to mature SoxR, we used a chromosomal P*soxS*::*lacZ* fusion at the *lac* locus, and β-galactosidase level as a proxy for monitoring SoxR maturation efficiency [33]. In the wt strain, induction of the P*soxS*::*lacZ* fusion reached a plateau at 180 min after adding PMS (30 μM) (Fig 5A). The β-galactosidase level in the treated strain was 22-fold higher than in the untreated cells (Fig 5A). In the Δ*soxR* strain, no induction of P*soxS*::*lacZ* expression was observed (Fig 5A). In the Δ*sufABCDSE* mutant, expression of the P*soxS*::*lacZ* plateaued *ca* 30 min after PMS addition (Fig 5B). The maximal level of β-galactosidase reached in the Δ*sufABCDSE* mutant was 2.5-fold lower than in the wt strain. In the Δ*iscUA* mutant, at all time points, the induction pattern of P*soxS*::*lacZ* was identical to the one observed in the wt strain (Fig 5C). All these results suggest that during the 30 min period after PMS exposure, the existing pool of [2Fe-2S]-bound SoxR was sufficient to activate *soxS* expression, in either Δ*sufABCDSE* or Δ*iscUA* mutants. In contrast, after 30 min SoxR-mediated activation occurred only in SUF containing strains. It is interesting to note that in the Δ*sufABCDSE* mutant, SoxR activity ceased shortly before growth stopped. It is tempting to speculate that the former is the cause of the latter (Fig 2A and Fig 5B).

**Fig 6:**
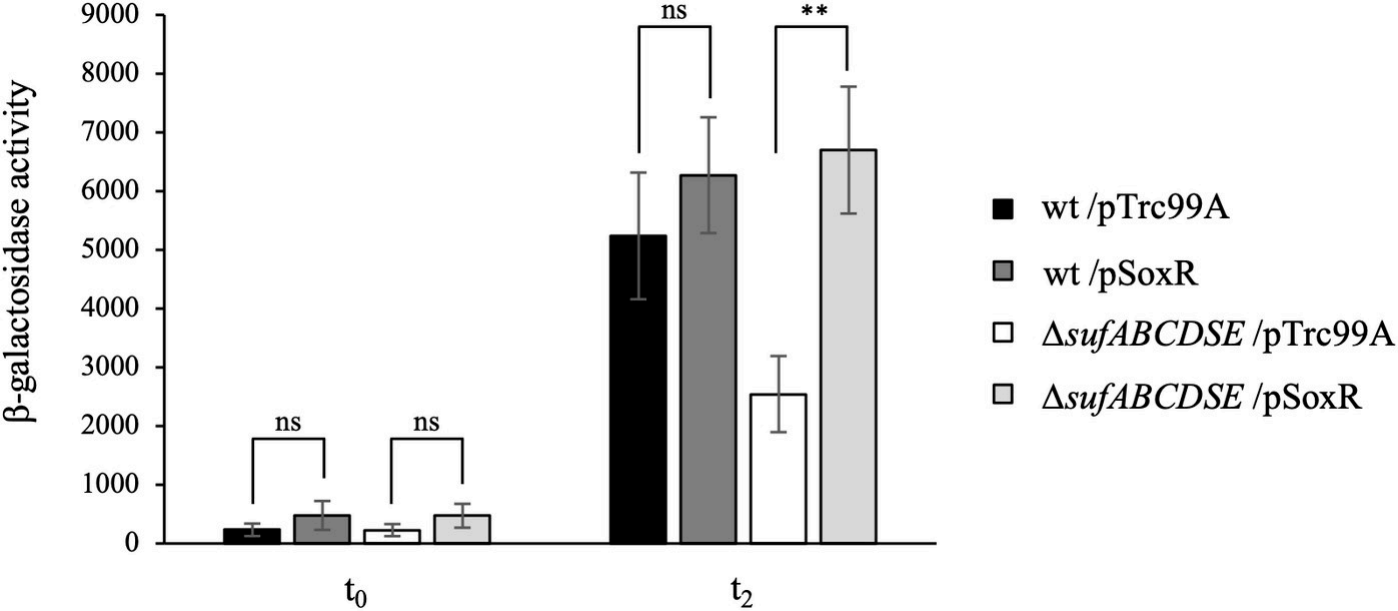
Overexpression of SoxR in the Δ*sufABCDSE* mutant. The *E*. *coli* wt (BE1000) and Δ*sufABCDSE* (AG031) strains carrying the chromosomal P*soxS*::*lacZ* fusion and the pSoxR plasmid or the empty plasmid (pTrc99A) were grown in LB supplemented with IPTG (0.1 mM) until OD_600_ reached 0.2. At time zero (t_0_), the cultures were treated with PMS (30 μM), and β-galactosidase activity was assayed at t_0_ and after 2 hours (t_2_) of growth and expressed as Miller units. The experiments were repeated at least three times. The means and standard deviations are shown. Asterisks represent the statistical significance calculated using the Bonferroni method (** p<0.01; * p<0.05; ns p>0.05).

We then analyzed the correlation between PMS concentration and induction of the SoxRS regulon in the different genetic backgrounds. Expression of the P*soxS*::*lacZ* fusion was measured 2 hours after incubation with different concentrations of PMS (3.4, 6.8, 10, 20 and 30 μM). At low PMS concentrations, *i*.*e*. 3.4 μM and 6.8 μM, P*soxS*::*lacZ* expression was induced to the same extent in all wt, Δ*iscUA* and Δ*sufABCDSE* strains (Fig 5D). In contrast, at PMS concentrations of 10 μM and above, P*soxS*::*lacZ* expression was much stronger in the wt and Δ*iscUA* strains than in the Δ*sufABCDSE* mutant (Fig 5D). In fact, in the Δ*sufABCDSE* mutant, expression of P*soxS*::*lacZ* plateaued when using PMS concentrations of 10 μM and above (Fig 5D). Interestingly, defect in P*soxS*::*lacZ* fusion expression within the Δ*sufABCDSE* strain was suppressed by a high *soxR* gene dosage, consistent with the notion that SoxR is a poor substrate for ISC system and that increased SoxR level would compensate inefficient maturation by ISC (Fig 6).

**Fig 7:**
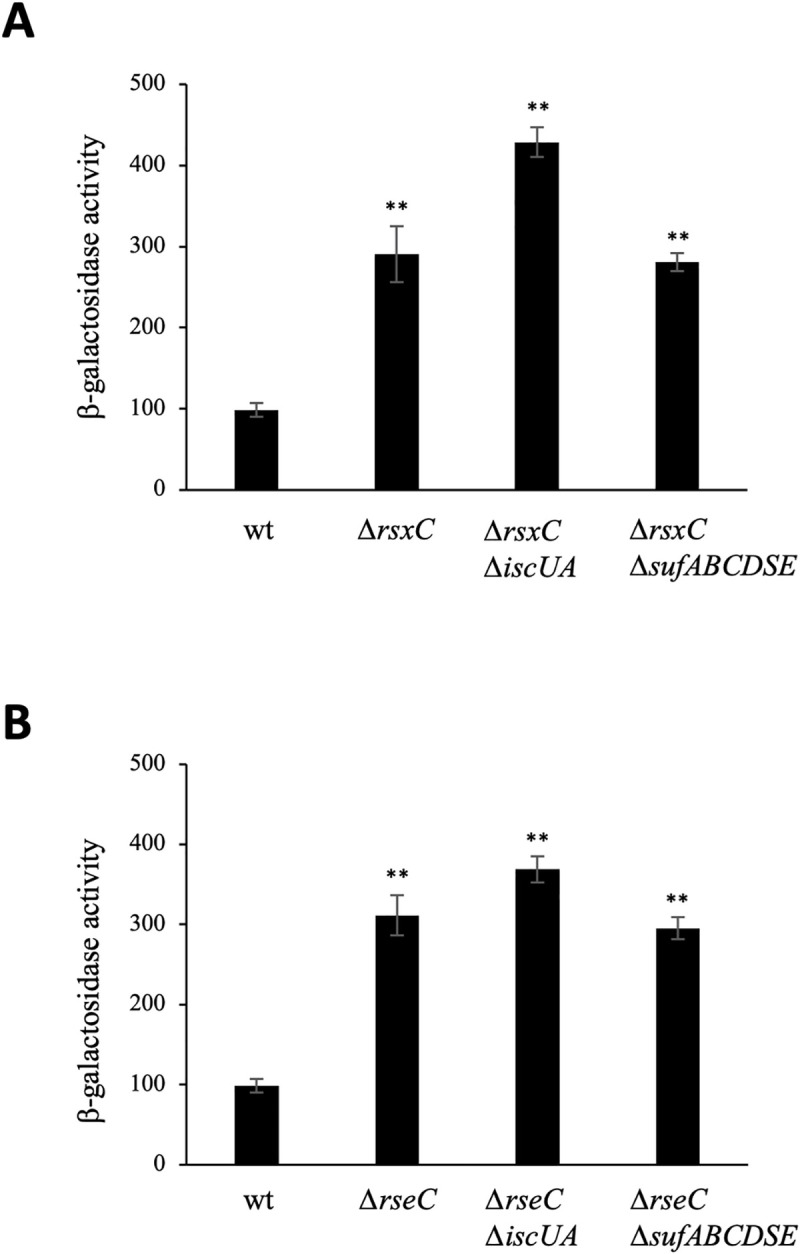
SoxR activity in mutants of the reduction system of SoxR. The *E*. *coli* strains carrying the chromosomal P*soxS*::*lacZ* fusion, wt (BE1000), Δ*rsxC* (AG047), Δ*rsxC* Δ*iscUA* (AG066), Δ*rsxC* Δ*sufABCDSE* (AG067), Δ*rseC* (AG045), Δ*rseC* Δ*iscUA* (AG064) and Δ*rseC* Δ*sufABCDSE* (AG065) were grown overnight in LB and inoculated (1/100) in fresh LB medium. The cultures were grown at 37°C until exponential phase (OD_600_ 0.2–0.4) (A-B). β-galactosidase activity was monitored and expressed as Miller units. The experiments were done in triplicate. The means and standard deviations are shown. The p-values have been determined to compare mutant strains versus the wt strain. Asterisks represent the statistical significance calculated using the Bonferroni method (** p<0.01; * p<0.05; ns p>0.05).

Last, we wished to investigate the contribution of ISC and SUF pathways to SoxR maturation in the absence of PMS. To this purpose we assessed the expression of the P*soxS*::*lacZ* fusion in two mutant strains, each lacking a component of the reduction system of SoxR, RsxC and RseC. In these strains, the Fe-S cluster of SoxR is thought to remain mostly oxidized and SoxR active even in the absence of redox-active molecules [34]. In the absence of PMS, activation of P*soxS*::*lacZ* transcription was increased (3-fold) in the Δ*rsxC* and Δ*rseC* mutants when compared to the wt strain (Fig 7). In the Δ*rsxC* Δ*iscUA*, Δ*rsxC* Δ*sufABCDSE*, Δ*rseC* Δ*iscUA* and Δ*rseC* Δ*sufABCDSE* double mutants, expression of the P*soxS*::*lacZ* fusion was increased to the same extent (3- to 4-fold) as compared to the wt strain (Fig 7). Together, these results indicate that under non-stressful conditions both Fe-S biogenesis systems, ISC and SUF, are able to sustain SoxR maturation.

Altogether, this series of results show that under non-stress inducing conditions, SoxR can acquire its cluster from either ISC or SUF system. However, under stress inducing conditions, SUF is the system that targets Fe-S cluster to SoxR, which subsequently get oxidized and permits SoxR to activate expression of its targets.

### The role of SoxR maturation in PMS/fluoroquinolone antagonism

We asked whether different levels of induction of the SoxRS regulon would translate in different level of norfloxacin resistance. Results showed that a concentration of 3.4 μM PMS was not sufficient to confer norfloxacin resistance to any of the strains under study (S3 Fig). At 30 μM PMS concentration, wt and Δ*iscUA* strains both reached similar OD_600_ whether or not norfloxacin was added (S3 Fig). In contrast, in the Δ*sufABCDSE* mutant, addition of norfloxacin impeded growth even in the presence of PMS (S3 Fig). Interestingly, at 30 μM PMS, expression of the P*soxS*::*lacZ* fusion was higher in the wt and Δ*iscUA* strains than in the Δ*sufABCDSE* mutant. These results establish the key role of SUF in allowing high PMS-mediated induction of SoxR, which eventually confers enhanced resistance to norfloxacin.

### SufA is required for SoxR maturation during PMS stress

**Fig 8:**
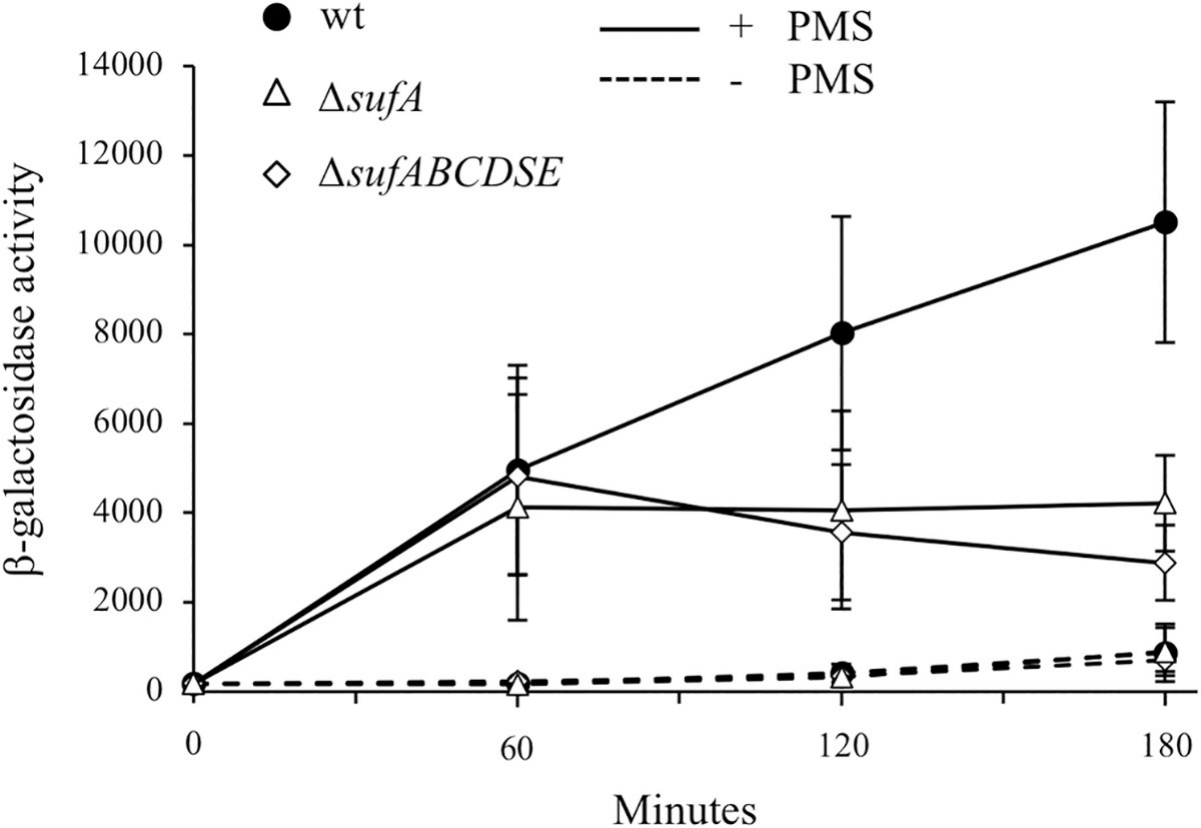
Maturation of SoxR requires the Fe-S clusters carrier, SufA, during PMS stress. The *E*. *coli* strains carrying the chromosomal P*soxS*::*lacZ* fusion, wt (BE1000) (black circles), Δ*sufA* (AG069) (white triangles) and Δ*sufABCDSE* (AG031) (white diamonds) were grown overnight in LB and inoculated (1/100) in fresh LB medium. The cultures were grown to an OD_600_ of 0.2, and were each split into two flasks, PMS (30 μM) was added (time zero) in one (solid line), and the other was left untreated (dotted line). All cultures were further incubated at 37°C with shaking and β-galactosidase activity was monitored and expressed as Miller units. The experiments were done in triplicate. The means and standard deviations are shown.

Once formed within the scaffold component of the Fe-S clusters biogenesis pathway, clusters are targeted to apo-protein clients *via* a series of carriers, which might show some level of redundancy depending upon the growth conditions [35,36]. Therefore, contribution of carriers in SoxR maturation was investigated. We showed above that expression of the P*soxS*::*lacZ* fusion was identical in the Δ*iscUA* mutant and in the wt strain, ruling out a role for IscA in SoxR maturation under PMS stress (Fig 5C). In the Δ*sufA* mutant, expression of the P*soxS*::*lacZ* fusion was identical to the wt strain after the first hour of incubation with PMS (Fig 8). In contrast, after longer exposure to PMS (2 and 3 hours), expression of the P*soxS*::*lacZ* fusion was drastically reduced when compared to the wt strain. Actually, the profile of expression of the P*soxS*::*lacZ* fusion was identical in the Δ*sufA* and in the Δ*sufABCDSE* mutants (Figs 5B and 8). These results indicate that SufA is required for SoxR maturation upon PMS stress.

### The stress responding carriers, NfuA and ErpA, are dispensable for SoxR maturation during PMS stress

NfuA and ErpA are additional Fe-S carriers that can cooperate under oxidative stress conditions [37–39]. Moreover, they are able to receive Fe-S clusters made within either ISC or SUF systems [37–39]. Given that SoxR is a stress responding regulator, it was of interest to evaluate the role of ErpA and NfuA in SoxR maturation. The expression profile of the P*soxS*::*lacZ* fusion upon PMS treatment was similar in the Δ*nfuA* and in the wt strains (S4 Fig) indicating that NfuA was not required for SoxR maturation.

The *erpA* gene is an essential gene in *E*. *coli* [38]. Therefore a conditional allele, in which the endogenous *erpA* gene is under the control of the P*araBAD* promoter (P*araBAD*::*erpA*), was transduced in the P*soxS*::*lacZ* fusion-containing strain. Strikingly, whether cells were grown in glucose or in arabinose, PMS induction of the P*soxS*::*lacZ* fusion was observed in the wt and P*araBAD*::*erpA* strains, indicating that ErpA was dispensable for SoxR maturation (S5 Fig panels A and B). To confirm the dispensability of ErpA for SoxR maturation, we used the CRISPR interference method to control *erpA* expression [40]. For this purpose, we used a plasmid producing a catalytically inactive version of Cas9 (pdCas9) and a plasmid encoding a single guide RNA (pRBS-*erpA*) targeting the non-template DNA strand of the UTR of *erpA* containing the ribosome-binding site. In the presence of the inducer anhydrotetracycline (aTc), cells carrying the pRBS-*erpA* exhibited a growth defect (S5 Fig panel C). In the presence of both aTc and PMS, P*soxS*::*lacZ* expression was the same in wt cells carrying the pRBS-*erpA* or the control vector, psgRNA (S5 Fig panel E). To verify that the pRBS-*erpA* plasmid was indeed preventing *erpA* expression, we used the strain PM2040 that contained an P*erpA*::*lacZ* gene fusion. We showed that in the presence of aTc, expression of P*erpA*::*lacZ* was almost null (2 Miller units) in cells carrying the pRBS-*erpA*, while β-galactosidase activity of 36 Miller units was measured in cells carrying control vector psgRNA (S5 Fig panel D). Altogether, results obtained by two different methods to deplete ErpA led us to conclude that ErpA is not required for SoxR maturation under PMS stress.

## Discussion

Understanding the influence of environmental conditions on level of antibiotic resistance is a prerequisite to monitor and control bacterial antibiotic resistance. Previously, we showed that iron limitation enhanced level of resistance of *E*. *coli* to aminoglycosides, and that Fe-S cluster biogenesis regulation played a key role in this unexpected link [8,41]. Here we show that Fe-S homeostasis connects ROS producing compound, RCC, and resistance level to fluoroquinolones.

*E*. *coli* synthesizes *ca*. 150 Fe-S proteins, the maturation of which depends upon ISC or SUF machineries. *E*. *coli* synthesizes five Fe-S bound transcriptional regulators, namely FNR, NsrR, IscR, YeiL, and SoxR [16,42]. Study of maturation of NsrR and IscR, which sense NO and Fe-S cluster demand, respectively, showed that these two related targets (*i*.*e*. *ca*. 40% sequence identity) are matured by ISC under normal conditions and by SUF under stress conditions [35]. In contrast, work by Kiley’s lab showed that ISC, but not SUF, was responsible for the maturation of FNR, a Fe-S transcriptional regulator sensing anaerobic/aerobic switch [43]. Here, we found SoxR to be matured mostly by SUF, a situation somehow complementary to that observed with FNR. SoxR being matured by SUF is consistent with the fact that SUF is synthesized and functional *in vivo* under oxidative stress [30,31,44–46].

The question then arises of what prevents ISC to act on SoxR. Previous *in vitro* transcription analyses showed that expression of both *isc* and *suf* operons is induced by PMS, which we confirmed here using *lacZ* fusions (S1 Fig) [30]. This rules out the possibility that defect in SoxR maturation was due to differential expression of the *isc* and *suf* operons. Increased copy number of the *soxR* gene suppressed the defect in *soxS* activation of the Δ*sufABCDSE* mutant, consistent with the idea that SoxR can be a low affinity substrate for ISC under PMS stress. Note however that SoxR protein levels may be lowered in a Δ*sufABCDSE* mutant as SoxR is positively autoregulated. Yet, use of the Δ*rsxC* and Δ*rseC* mutants allowed us to show that in the absence of PMS, ISC is able to maturate SoxR. Hence, we propose that under PMS stress, SoxR is a poor substrate for ISC because ISC system itself is intrinsically susceptible to oxidative stress, possibly as a result of PMS-mediated damages to some of the Isc proteins. That SoxR can be maturated by both machineries is consistent with the fact that SoxR orthologs can be found in bacterial species that have only SUF such as *Streptomyces coelicor*, or in bacterial species that have only ISC, such as *Pseudomonas aeruginosa* [24,47,48].

During the delivery step, Fe-S clusters are transferred from the ISC or SUF systems to the apo-targets. Multiple studies have concluded that ErpA carrier is the ultimate carrier used by most, if not all, cellular proteins, including IspG/H, formate dehydrogenase N, nitrate reductase, succinate dehydrogenase, complex I, or hydrogenases 1 and 2 [37,38,49–51]. Surprisingly, in the present study, maturation of SoxR was found totally independent of ErpA. SoxR maturation was also independent of the ErpA-NfuA delivery pathway we recently identified as being important for combating oxidative stress [37]. This series of observation was totally unexpected and opens new perception of Fe-S biogenesis under stress conditions. An explanation for the ErpA/NfuA independent maturation of SoxR might be that it binds a 2Fe-2S cluster, whereas all other proteins tested so far bind 4Fe-4S clusters (with the noticeable exception of IscR). That delivery factors such as A-type carriers (IscA, SufA, ErpA) be required for 4Fe-4S containing targets was already suggested by our study comparing IscR and NsrR maturation maps, and was also proposed on the basis of the *in vivo* maturation of the 2Fe-2S containing ferredoxin Fdx and from *in vivo* studies in yeast [35,52,53]. Altogether, our study is fully consistent with the pioneer work of Schwartz and collaborators [54], which demonstrated that IscS was not needed for SoxR maturation under paraquat stress. However, the results presented here go far beyond this original finding in describing both the basic machinery and the accessory proteins that transfer the cluster from the scaffold to SoxR.

We previously reported that *E*. *coli* making clusters with SUF showed an enhanced resistance to aminoglycoside and here we report that it also shows an enhanced resistance to fluoroquinolone [8]. Beyond this apparent similarity, opposite molecular causes are found. Because the SUF system is *less efficient* than the ISC for maturating pmf-producing Fe-S cluster containing Nuo and Sdh respiratory complexes, uptake of aminoglycoside was reduced and bacteria exhibited aminoglycoside phenotypic resistance. On the contrary, under RCC stress, because the SUF system is *more efficient* than the ISC one for maturating SoxR, cells can expel fluoroquinolone and show enhanced fluoroquinolone resistance. An enigma then remains: AcrAB-mediated fluoroquinolone efflux being itself energized by pmf, fluoroquinolone (and possibly PMS) efflux by AcrAB should have been reduced in PMS-treated cells that should have been scored as hypersensitive instead of hyperresistant. Measuring the actual drop in pmf induced by DIP and by PMS, and assessing how much pmf is required for AG uptake and fluoroquinolone efflux will probably help to solve this apparent conundrum.

Drug/drug interaction might mimic situations in natural settings wherein bacteria face multiple antibacterial chemicals. In the case of DIP/aminoglycoside antagonism, one could speculate that iron limitation can be interpreted as a signal to adapt to a hostile environment, by reducing all pmf-dependent exchanges. RCC and fluoroquinolone share structural similarity as they are both heterocyclic compounds and could be present within the same ecological niche. In this regard, *Pseudomonas aeruginosa* provides a good illustration of evolved adaptation to such compounds: the MexGHI-OpmD pump excretes both fluoroquinolone and 5-methylphenazine-1-carboxylate, an intermediate of pyocyanin biosynthesis, which is structurally similar to PMS and activates the *mexGHI-opmD* operon that belongs to the SoxR regulon [55–58]. As a matter of fact, pyocyanin was also found to antagonize activity of many types of antibiotics including fluoroquinolone [5,59]. Thus, a possibility is that in *E*. *coli*, the fluoroquinolone exporting AcrAB pump will export PMS as well. The very low MIC_PMS_ value of *acrA* mutant supports this view. Very recently, the RCC/fluoroquinolone antagonism was shown to be conserved in *P*. *aeruginosa*, and importantly it occurred in *P*. *aeruginosa* biofilms that are an important cause for persistent and antibiotics-resistant infections [5,60]. Altogether these results illustrate the medical importance of the RCC/fluoroquinolone antagonism. As a last note, a recent system-based analysis predicted ROS as potential adjuvants potentiating antibacterial activity [61]. On the contrary, the present and previous studies identified antagonism between ROS producers RCC and several quinolones (norfloxacin, ciprofloxacin, levofloxacin, novobiocin and moxifloxacin). Hence, one should be cautious when using ROS producing chemical as antibiotic adjuvant.

## Materials and methods

### Bacterial strains and growth conditions

The transcriptional P*soxS*::*lacZ* fusion was constructed as described in Ezraty *et al*. [33]. The P*soxS* promoter region fused to *lacZ* encompassed a region from the 111 nucleotides upstream the transcriptional *soxS* start site to the 21 first nucleotides of the *soxS*-coding region. The *E*. *coli* K-12 strain MG1655 and its derivatives used in this study are listed in S1 Table. Deletion mutations were introduced by P1 transduction. Transductants were verified by PCR, using primer pairs hybridizing upstream and downstream of the deleted gene. *E*. *coli* strains were grown at 37°C in Luria-Bertani (LB) rich medium. Isopropyl β-D-1-thiogalactopyranoside (IPTG) (0.1 mM), arabinose (0.2%), glucose (0.2%) and anhydrotetracycline (aTc) (2 μM) were added when required. Solid media contained 1.5% agar. Antibiotics were used at the following concentrations: chloramphenicol 25 μg/mL, kanamycin 30 μg/mL, and ampicillin 50 μg/mL.

### MIC determination

To determine MIC_PMS_, PMS was dissolved in LB medium and diluted in LB to reach concentration ranging from 0 to 300 μM in 20 μM increments. One hundred microliters (100 μL) of each concentration of PMS tested were added in a 96-well microplate. Each well was inoculated with 100 μL of a fresh LB bacterial inoculum of 2 × 10^5^ c.f.u./mL, obtained from a dilution of a mid-log phase *E*. *coli* growing culture. To determine MIC_Norfloxacin_ of strains carrying the pSoxS and pTrc99A plasmids, norfloxacin was dissolved in LB medium and diluted in LB to reach concentration ranging from 800 ng/mL to 1800 ng/mL in 100 ng/mL increments for the strains carrying the pSoxS, and 160 ng/mL to 360 ng/mL in 20 ng/mL increments for the strains carrying the pTrc99A. One hundred microliters (100 μL) of each concentration of norfloxacin tested were added in a 96-well microplate. Each well was inoculated with 100 μL of a fresh LB (supplemented with ampicillin and IPTG) bacterial inoculum of 2 × 10^5^ c.f.u./mL, obtained from a dilution of a mid-log phase *E*. *coli* growing culture. Ampicillin and IPTG were added to the LB medium used to dilute the *E*. *coli* culture. Microplates were incubated at 37°C for 18 h under aerobic conditions and agitation 170 rpm. The microplates were then read at OD_600nm_ and MIC was defined as the lowest drug concentration that exhibited complete inhibition of *E*. *coli* growth. The experiment was repeated at least three times.

### PMS-mediated protection against norfloxacin

Wells of a 96-well microplate containing 100 μL of LB supplemented or not with norfloxacin and PMS, were inoculated with 100 μL of a fresh LB bacterial inoculum of 2 × 10^5^ c.f.u./mL. The range of norfloxacin final concentrations used was adapted to each strain depending on their sensitivity, and is given in the corresponding figures. PMS was used at the indicated final concentration. Microplates were incubated at 37°C for 18 h under aerobic conditions and agitation (170 rpm). The microplates were then read at OD_600nm_.

### Plasmid construction

Plasmids pSoxR and pSoxS were constructed by PCR amplification of the coding region of *soxR* and *soxS* from *E*. *coli* MG1655 chromosomal DNA using the following primer pair: *Nco*I-*soxR*/*BamH*I-*soxR* and *Nco*I-*soxS*/*BamH*I-*soxS*, respectively (S2 Table). The PCR product was then digested by *Nco*I and *BamH*I and cloned into the *Nco*I/*BamH*I linearized pTrc99A vector. The plasmids were verified by sequencing. The pRBS-*erpA* plasmid was cloned as described by Larson *et al*. [40]. Briefly, we designed primers hybridizing at *erpA* RBS region (Ec-F and Ec-R), which were used to PCR-amplify the whole plasmid (psgRNA). The plasmid was verified by sequencing using primers Ec-F colony and Ec-R colony. The sgRNA obtained was complementary to the *erpA* non-template strand.

### RNA preparation and reverse transcription

The *E. coli* wt strain (BE1000) was grown in LB. When the culture reached mid-exponential phase, the culture was divided in four aliquots, three were treated for 30 min with norfloxacin only (30 ng/mL), with PMS only (30 μM), or with both norfloxacin and PMS, while the last sample remained untreated. Three biological independent experiments were performed. RNAs were prepared from *E*. *coli* strain cultures (10 mL) grown in appropriate conditions. The cells were harvested and frozen at -80°C. Total RNAs were isolated from the pellet using the Maxwell 16 LEV miRNA Tissue Kit (Promega) according to the manufacturer’s instructions and an extra TURBO DNase (Invitrogen) digestion step to eliminate the contaminating DNA. The RNA quality was assessed by tape station system (Agilent). RNA was quantified spectrophotometrically at 260 nm. For cDNA synthesis, 1 μg total RNA and 0.5 μg random primers were used with the GoScript Reverse transcriptase according to the manufacturer instruction (Promega).

### Quantitative real-time-PCR for transcriptional analyses

Quantitative real-time PCR (qPCR) analyses were performed on a CFX96 Real-Time System (Bio-Rad). The reaction volume was 15 μL and the final concentration of each primer was 0.5 μM. The cycling parameters of the qRT-PCR were 98°C for 2 min, followed by 45 cycles of 98°C for 5 s, 55°C for 10 s, 72°C for 1 s. A final melting curve from 65°C to 95°C is added to determine the specificity of the amplification. To determine the amplification kinetics of each product, the fluorescence derived from the incorporation of EvaGreen into the double-stranded PCR products was measured at the end of each cycle using the SsoFast EvaGreen Supermix 2X Kit (Bio-Rad). The results were analyzed using Bio-Rad CFX Maestro software, version 1.1 (Bio-Rad). The RNA16S gene was used as a reference for normalization. For each point a technical duplicate was performed. The amplification efficiencies for each primer pair were comprised between 80 and 100%. The primers used for qRT-PCR are reported in the S2 Table.

### β-Galactosidase assay

β-Galactosidase assays were carried out as described previously by J.H. Miller [62].

### Test for *E*. *coli* sensitivity to redox-cyclic compounds

The *E*. *coli* strains were grown overnight in LB and inoculated (1/100) in fresh LB medium. The cultures were grown to an OD_600_ of 0.2, and were each split into two flasks, one with PMS added (time zero), while the other was left untreated. Cultures were further incubated at 37°C and growth was monitored by following OD_600_. To test the *E*. *coli* sensitivity to redox-cyclic compounds on plates, overnight cultures were diluted in sterile PBS and 5 μL were directly spotted onto LB plates containing PMS. The plates were incubated overnight at 37°C before growth was scored.

### ErpA depletion

The LL401 strain carrying the chromosomal copy of *erpA* gene under the P*araBAD* promoter [38] was grown overnight in LB. Fresh LB medium supplemented with glucose (0.2%) was then inoculated at 1/100. The strain carrying the plasmids allowing controlling *erpA* expression by CRISPRi, pdCas9 and pRBS-*erpA*, was grown overnight in LB and then inoculated (1/100) in fresh LB supplemented with anhydrotetracycline (aTc) (2 μM).

## Supporting information

S1 Table Strains and plasmids used in this study. (DOCX)

S2 Table Primers used in this study. (DOCX)

S1 Fig Expression of the *sufABCDSE* and *iscRSUA* operons during PMS stress. The *E*. *coli* strains carrying the chromosomal P*sufA*::*lacZ* fusion (PM2081) (A) or the P*iscR*::*lacZ* fusion (DV901) (B) were grown overnight in LB and inoculated (1/100) in fresh LB medium. Cultures were grown to an OD_600_ of 0.2 and split into two flasks, PMS (20 μM) was added (time zero) in one, and the other was left untreated. All cultures were further incubated at 37°C for 2 hours with shaking. β-galactosidase activity was monitored and expressed as Miller units. The experiments were repeated at least three times. The means and standard deviations are shown.
(DOCX)

S2 Fig PMS sensitivity in genetic backgrounds with enhanced endogenous superoxide stress. *E*. *coli* strains wt (MG1655), Δ*iscUA* (DV597), Δ*sufABCDSE* (BP198), Δ*sodA* (AG011), Δ*sodA* Δ*iscUA* (AG004), Δ*sodA* Δ*sufABCDSE* (AG006), Δ*sodB* (BE258), Δ*sodB* Δ*iscUA* (AG005), Δ*sodB* Δ*sufABCDSE* (AG007), Δ*sodA* Δ*sodB* (BE259), Δ*sodA* Δ*sodB* Δ*iscUA* (AG024), and Δ*sodA* Δ*sodB* Δ*sufABCDSE* (AG025) were grown in LB until OD_600_ reached 0.2, then serially diluted in PBS and spotted on LB plates containing or not PMS at the indicated concentrations. Each spot represents a 10-fold serial dilution of the bacterial culture. Plates were read after overnight incubation at 37°C. The experiment was repeated at least three times. One representative experiment is shown.
(DOCX)

S3 Fig PMS-dependent induction of the P*soxS*::*lacZ* fusion and norfloxacin resistance. The *E*. *coli* wt (BE1000), Δ*iscUA* (AG030), and Δ*sufABCDSE* (AG031) strains were grown to mid-log phase in LB and then diluted to inoculate 96-well microplate wells containing liquid LB medium (grey bars), LB medium supplemented with PMS (3.4 μM in panel A; 30 μM in panel B) (white bars), LB medium supplemented with norfloxacin (160 ng/mL) (hatched bars), and LB medium supplemented with both PMS and norfloxacin (160 ng/mL) (black bars). Cultures were incubated 18 hours at 37°C with shaking. Plates were read for OD_600_ in Tecan Infinite. The experiments were repeated at least three times. The means and standard deviations are shown.
(DOCX)

S4 Fig NfuA is dispensable for SoxR maturation during PMS stress. The *E*. *coli* strains carrying the chromosomal P*soxS*::*lacZ* fusion, wt (BE1000) (black circles) and Δ*nfuA* (AG043) (white squares) were grown in LB until OD_600_ reached 0.2. At time zero, the cultures were treated with PMS (30 μM), and β-galactosidase activity was monitored and expressed as Miller units. The experiments were repeated at least three times. The means and standard deviations are shown.
(DOCX)

S5 Fig ErpA is not required for SoxR maturation. (A-B) The *E*. *coli* strains carrying the chromosomal P*soxS*::*lacZ* fusion, wt (BE1000) (black circles) and P*araBAD*::*erpA* (AG048) (black crosses) (A-B), were grown overnight in LB and then inoculated (1/100) in fresh LB medium with glucose (0.2%) (A) or arabinose (0.2%) (B). The cultures were grown to an OD_600_ of 0.2, and were each split into two flasks, PMS (30 μM) was added (time zero) in one (solid line), and the other was left untreated (dotted line). All cultures were further incubated at 37°C with shaking. β-galactosidase activity was monitored and expressed as Miller units. (C) Growth of the *E*. *coli* wt strain (BE1000) containing the chromosomal P*soxS*::*lacZ* fusion and carrying the plasmids pdCas9 together with the plasmid allowing *erpA* extinction, pRBS-*erpA* (white triangles), or the empty control vector, psgRNA (black circles). Cells were grown overnight in LB without anhydrotetracycline (aTc) and then inoculated (1/100) in fresh LB medium supplemented with aTc (2 μM). Growth was recorded by measuring OD_600_ following time. (D) The *E*. *coli* strain possessing the chromosomal P*erpA*::*lacZ* fusion (PM2040) and carrying the plasmid pdCAS9 together with the plasmid allowing *erpA* extinction, pRBS-*erpA* or the control vector, psgRNA. Cells were grown overnight in LB without anhydrotetracycline (aTc) and then inoculated (1/100) in fresh LB medium supplemented with aTc (2 μM). When cultures reached an OD_600_ of 0.2, aliquots were taken to assay β-galactosidase activity that is expressed in Miller units. (E-F) The *E*. *coli* wt strain possessing the chromosomal P*soxS*::*lacZ* fusion (BE1000) and carrying the plasmid pdCas9 together with either the plasmid allowing *erpA* extinction, pRBS-*erpA* (white triangles), or the empty control vector, psgRNA (black circles) were grown overnight in LB and then inoculated (1/100) in fresh LB medium supplemented (E) or not (F) with aTc (2 μM). The cultures were grown to an OD_600_ of 0.2, and were each split into two flasks, PMS (30 μM) was added (time zero) in one (solid line), and the other was left untreated (dotted line). All cultures were further incubated at 37°C with shaking and β-galactosidase activity was monitored and expressed as Miller units. All the experiments were repeated at least three times. The means and standard deviations are shown (A, B, D, E and F) and a representative experiment is presented in panel C.
(DOCX)
