# Supplementary material for: Oxidative stress antagonizes fluoroquinolone drug sensitivity via the SoxR-SUF Fe-S cluster homeostatic axis
Source: PLoS Genet. 2020 Nov 2;16(11):e1009198. doi: 10.1371/journal.pgen.1009198 (PMC7671543; doi:10.1371/journal.pgen.1009198)
Supplement: S1 Table — (DOCX) [file pgen.1009198.s001.docx]

**S1 Table. Strains and plasmids used in this study.**

___________________________________________________________________________

Strain Relevant genotype Source

___________________________________________________________________________

BE101 MG1655 parental strain Lab collection

YD002 MG1655 ∆*micF::kan* This study

YD001 MG1655 ∆*acrA::kan* This study

BE1000 MG1655 derivative *PsoxS ::lacZ* [1]

AG030 BE1000 derivative ∆*iscUA::cat* This study

AG031 BE1000 derivative ∆*sufABCDSE::cat* This study

AG035 BE1000 derivative ∆*soxR::kan* This study

AG043 BE1000 derivative ∆*nfuA::kan* This study

AG069 BE1000 derivative ∆*sufA::kan* This study

AG047 BE1000 derivative ∆*rsxC::kan* This study

AG066 BE1000 derivative ∆*rsxC* (cured) ∆*iscUA::cat* This study

AG067 BE1000 derivative ∆*rsxC* (cured) ∆*sufABCDSE::cat* This study

AG045 BE1000 derivative ∆*rseC::kan* This study

AG064 BE1000 derivative ∆*rseC* (cured) ∆*iscUA::cat* This study

AG065 BE1000 derivative ∆*rseC* (cured) ∆*sufABCDSE::cat* This study

LL401 *erpA* gene under the control of the [2]

P*araBAD* promoter

AG048 BE1000 derivative carrying the chromosomic *erpA* This study

gene under the control of the P*araBAD* promoter

DV597 MG1655 Δ*iscUA*::*cat* [3]

BP198 MG1655 ∆*sufABCDSE*::*cat* [4]

AG011 MG1655 ∆*sodA*::*kan* This study

AG004 MG1655 ∆*sodA*::*kan* ∆*iscUA*::*cat* This study

AG006 MG1655 ∆*sodA*::*kan* ∆*sufABCDSE*::*cat* This study

BE258 MG1655 ∆*sodB*::*kan* [5]

AG005 MG1655 ∆*sodB*::*kan* ∆*iscUA*::*cat* This study

AG007 MG1655 ∆*sodB*::*kan* ∆*sufABCDSE*::*cat* This study

BE259 MG1655 Δ*sodA::cat* Δ*sodB::kan* [5]

AG000 MG1655 Δ*sodA* (cured) Δ*sodB::kan* This study

AG024 MG1655 Δ*sodA* (cured) Δ*sodB::kan* ∆*iscUA::cat* This study

AG025 MG1655 Δ*sodA* (cured) Δ*sodB::kan* ∆*sufABCDSE::cat* This study

DV901 MG1655 ∆*lacZ* *PiscR::lacZ* [6]

PM1205 MG1655 *mal*::*lacI^q^,* Δ*araBAD,* [7] *lacI'*::P*araBAD-cat-sacB-lacZ, mini λ tet^R^*

PM2081 PM1205 derivative, P*sufA::lacZ lacZ* at the *lac* locus Lab collection

PM2040 PM1205 derivative, P*erpA::lacZ lacZ* at the *lac* locus [8]

___________________________________________________________________________

Plasmids Relevant characteristic Source

___________________________________________________________________________

pTrc99A Cloning vector with an hybrid *trp/lac* promoter [9]

pSoxS pTrc99A derivative carrying the *soxS* gene This study

pSoxR pTrc99A derivative carrying the *soxR* gene This study

pdCas9 Addgene: no. 44249 [10]

Encodes a defective Cas9 protein

psgRNA Addgene: no. 44251 [10]

Encodes a single guide RNA

pRBS-*erpA* Encodes a single guide RNA complementary to This study

non template strand of *erpA*

___________________________________________________________________________

1. Ezraty B, Henry C, Hérisse M, Denamur E, Barras F. Commercial Lysogeny Broth culture media and oxidative stress: a cautious tale. Free Radic Biol Med. 2014;74: 245–251. doi:10.1016/j.freeradbiomed.2014.07.010

2. Loiseau L, Gerez C, Bekker M, Ollagnier-de Choudens S, Py B, Sanakis Y, et al. ErpA, an iron sulfur (Fe S) protein of the A-type essential for respiratory metabolism in Escherichia coli. Proc Natl Acad Sci USA. 2007;104: 13626–13631. doi:10.1073/pnas.0705829104

3. Vinella D, Brochier-Armanet C, Loiseau L, Talla E, Barras F. Iron-sulfur (Fe/S) protein biogenesis: phylogenomic and genetic studies of A-type carriers. PLoS Genet. 2009;5: e1000497. doi:10.1371/journal.pgen.1000497

4. Nachin L, Loiseau L, Expert D, Barras F. SufC: an unorthodox cytoplasmic ABC/ATPase required for [Fe-S] biogenesis under oxidative stress. EMBO J. 2003;22: 427–437. doi:10.1093/emboj/cdg061

5. Ezraty B, Vergnes A, Banzhaf M, Duverger Y, Huguenot A, Brochado AR, et al. Fe-S cluster biosynthesis controls uptake of aminoglycosides in a ROS-less death pathway. Science. 2013;340: 1583–1587. doi:10.1126/science.1238328

6. Vinella D, Loiseau L, Ollagnier de Choudens S, Fontecave M, Barras F. In vivo [Fe-S] cluster acquisition by IscR and NsrR, two stress regulators in Escherichia coli. Mol Microbiol. 2013;87: 493–508. doi:10.1111/mmi.12135

7. Mandin P, Gottesman S. A genetic approach for finding small RNAs regulators of genes of interest identifies RybC as regulating the DpiA/DpiB two-component system. Mol Microbiol. 2009;72: 551–565. doi:10.1111/j.1365-2958.2009.06665.x

8. Mandin P, Chareyre S, Barras F. A Regulatory Circuit Composed of a Transcription Factor, IscR, and a Regulatory RNA, RyhB, Controls Fe-S Cluster Delivery. MBio. 2016;7. doi:10.1128/mBio.00966-16

9. Amann E, Ochs B, Abel KJ. Tightly regulated tac promoter vectors useful for the expression of unfused and fused proteins in Escherichia coli. Gene. 1988;69: 301–315. doi:10.1016/0378-1119(88)90440-4

10. Larson MH, Gilbert LA, Wang X, Lim WA, Weissman JS, Qi LS. CRISPR interference (CRISPRi) for sequence-specific control of gene expression. Nat Protoc. 2013;8: 2180–2196. doi:10.1038/nprot.2013.132
