# Supplementary material for: Oxidative stress antagonizes fluoroquinolone drug sensitivity via the SoxR-SUF Fe-S cluster homeostatic axis
Source: PLoS Genet. 2020 Nov 2;16(11):e1009198. doi: 10.1371/journal.pgen.1009198 (PMC7671543; doi:10.1371/journal.pgen.1009198)
Supplement: S2 Table — (DOCX) [file pgen.1009198.s002.docx]

**S2 Table. Primers used in this study.**

___________________________________________________________________________

Primers for *soxR* and *soxS* cloning

***Nco*I *soxR***  5' GGCCCCATGGAAAAGAAATTACCCCG 3'

***Bam*HI *soxR*** 5' GCGCGGATCCTTAGTTTTGTTCATCTTCCAGCA 3'

***Nco*I *soxS***  5' GGGCCATGGCGTCCCATCAGAAAATTAT TCAG 3'

***Bam*HI *soxS*** 5' CCCGGATCCTTACAGGCGGTGGCGATAATC 3'

Primers for pRBS-*erpA* construction

**Ec-F** 5' TTTGCTCCAAACGACATCGGCAATTGTTTTAGAGCTAGAAATAGCAAGTTAAAATAAGGC 3’

**Ec-R** 5' ACTAGTATTATACCTAGGACTGAGCTAGC 3'

**Ec-F colony** 5' GGGTTATTGTCTCATGAGCGGATACATATTTG 3'

**Ec-R colony** 5' CGCGGCCTTTTTACGGTTC 3'

Primers for qRT-PCR

***acrA-Fw*** 5' TCAAAGAAGGTAGCGACATC 3'

***acrA-Rv*** 5' GACTTTGGTGTAAGCCAGAT 3'

***acrB-Fw*** 5' TTCTCGCAAATCAAAGATGC 3'

***acrB-Rv*** 5' TACGCTGGTCAACATATCAG 3'

___________________________________________________________________________
