# Supplementary material for: Oxidative stress antagonizes fluoroquinolone drug sensitivity via the SoxR-SUF Fe-S cluster homeostatic axis
Source: PLoS Genet. 2020 Nov 2;16(11):e1009198. doi: 10.1371/journal.pgen.1009198 (PMC7671543; doi:10.1371/journal.pgen.1009198)
Supplement: S1 Fig — The E. coli strains carrying the chromosomal PsufA::lacZ fusion (PM2081) (A) or the PiscR::lacZ fusion (DV901) (B) were grown overnight in LB and inoculated (1/100) in fresh LB medium. Cultures were grown to an OD600 of 0.2 and split into two flasks, PMS (20 μM) was added (time zero) in one, and the other was left untreated. All cultures were further incubated at 37°C for 2 hours with shaking. β-galactosidase activity was monitored and expressed as Miller units. The experiments were repeated at least three times. The means and standard deviations are shown. (DOCX) [file pgen.1009198.s003.docx]

**
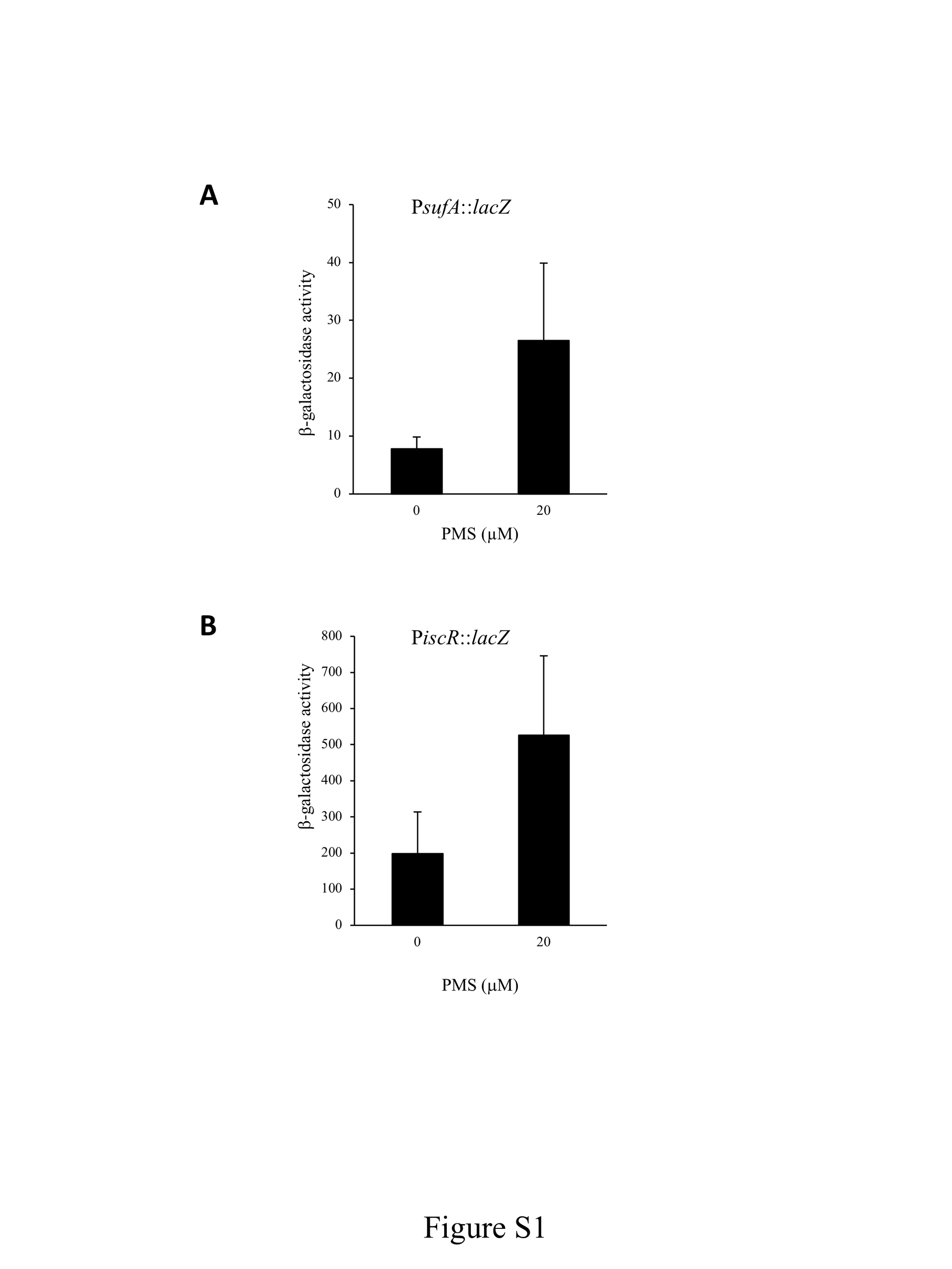
S1 Fig. Expression of the *sufABCDSE* and *iscRSUA* operons during PMS stress**

The *E. coli* strains carrying the chromosomal P*sufA::lacZ* fusion (PM2081) (A) or the P*iscR::lacZ* fusion (DV901) (B) were grown overnight in LB and inoculated (1/100) in fresh LB medium. Cultures were grown to an OD_600_ of 0.2 and split into two flasks, PMS (20 µM) was added (time zero) in one, and the other was left untreated. All cultures were further incubated at 37°C for 2 hours with shaking. β-galactosidase activity was monitored and expressed as Miller units. The experiments were repeated at least three times. The means and standard deviations are shown.
