# Supplementary material for: Oxidative stress antagonizes fluoroquinolone drug sensitivity via the SoxR-SUF Fe-S cluster homeostatic axis
Source: PLoS Genet. 2020 Nov 2;16(11):e1009198. doi: 10.1371/journal.pgen.1009198 (PMC7671543; doi:10.1371/journal.pgen.1009198)
Supplement: S2 Fig — E. coli strains wt (MG1655), ΔiscUA (DV597), ΔsufABCDSE (BP198), ΔsodA (AG011), ΔsodA ΔiscUA (AG004), ΔsodA ΔsufABCDSE (AG006), ΔsodB (BE258), ΔsodB ΔiscUA (AG005), ΔsodB ΔsufABCDSE (AG007), ΔsodA ΔsodB (BE259), ΔsodA ΔsodB ΔiscUA (AG024), and ΔsodA ΔsodB ΔsufABCDSE (AG025) were grown in LB until OD600 reached 0.2, then serially diluted in PBS and spotted on LB plates containing or not PMS at the indicated concentrations. Each spot represents a 10-fold serial dilution of the bacterial culture. Plates were read after overnight incubation at 37°C. The experiment was repeated at least three times. One representative experiment is shown. (DOCX) [file pgen.1009198.s004.docx]

**
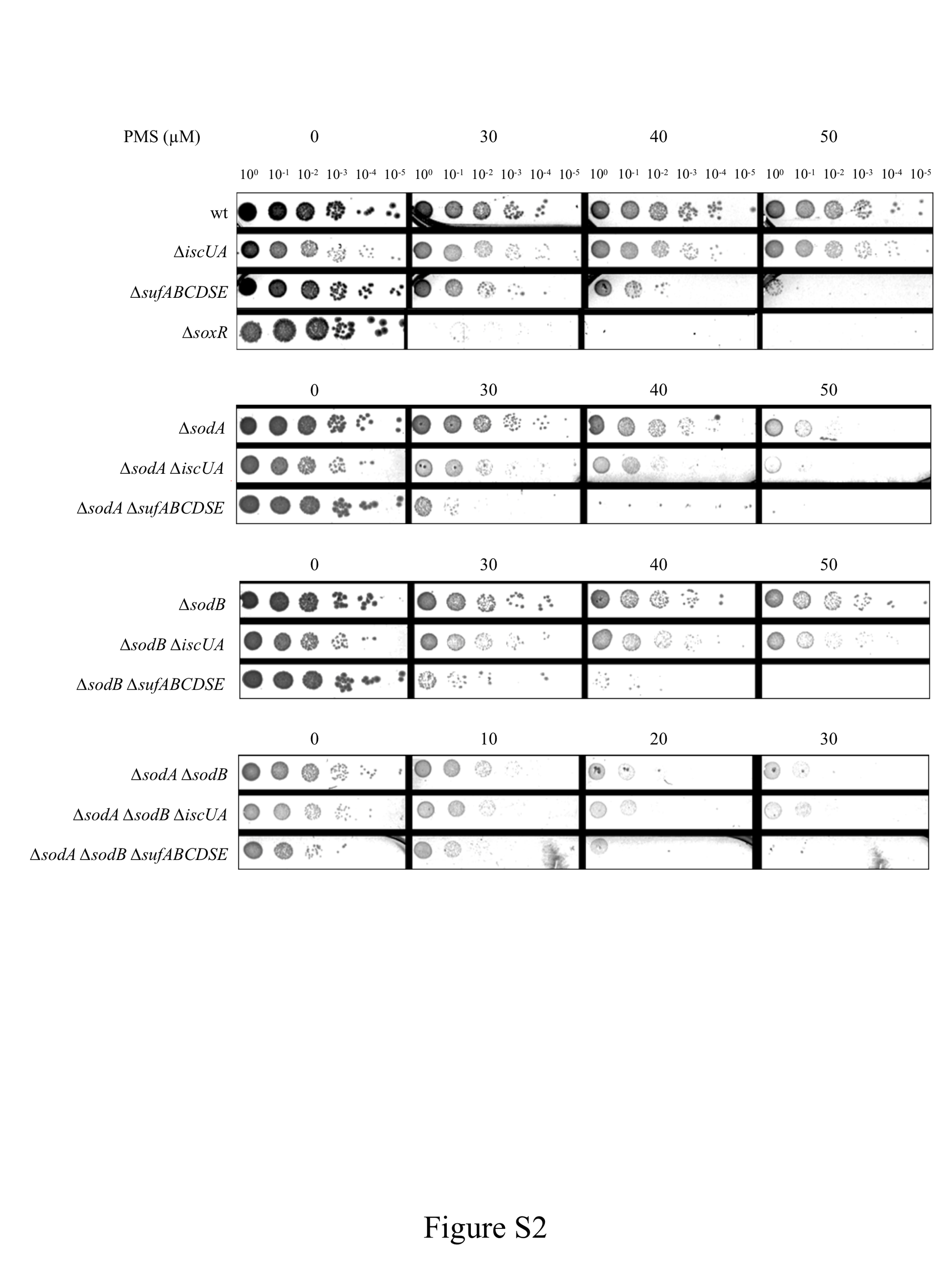
**

**S2 Fig. PMS sensitivity in genetic backgrounds with enhanced endogenous superoxide stress.**

*E. coli* strains wt (MG1655), ∆*iscUA* (DV597), ∆*sufABCDSE* (BP198), ∆*sodA* (AG011), ∆*sodA* ∆*iscUA* (AG004), ∆*sodA* ∆*sufABCDSE* (AG006), ∆*sodB* (BE258), ∆*sodB* ∆*iscUA* (AG005), ∆*sodB* ∆*sufABCDSE* (AG007), ∆*sodA* ∆*sodB* (BE259), ∆*sodA* ∆*sodB* ∆*iscUA* (AG024), and ∆*sodA* ∆*sodB* ∆*sufABCDSE* (AG025) were grown in LB until OD_600_ reached 0.2, then serially diluted in PBS and spotted on LB plates containing or not PMS at the indicated concentrations. Each spot represents a 10-fold serial dilution of the bacterial culture. Plates were read after overnight incubation at 37°C. The experiment was repeated at least three times. One representative experiment is shown.
