# Supplementary material for: Oxidative stress antagonizes fluoroquinolone drug sensitivity via the SoxR-SUF Fe-S cluster homeostatic axis
Source: PLoS Genet. 2020 Nov 2;16(11):e1009198. doi: 10.1371/journal.pgen.1009198 (PMC7671543; doi:10.1371/journal.pgen.1009198)
Supplement: S3 Fig — The E. coli wt (BE1000), ΔiscUA (AG030), and ΔsufABCDSE (AG031) strains were grown to mid-log phase in LB and then diluted to inoculate 96-well microplate wells containing liquid LB medium (grey bars), LB medium supplemented with PMS (3.4 μM in panel A; 30 μM in panel B) (white bars), LB medium supplemented with norfloxacin (160 ng/mL) (hatched bars), and LB medium supplemented with both PMS and norfloxacin (160 ng/mL) (black bars). Cultures were incubated 18 hours at 37°C with shaking. Plates were read for OD600 in Tecan Infinite. The experiments were repeated at least three times. The means and standard deviations are shown. (DOCX) [file pgen.1009198.s005.docx]

**
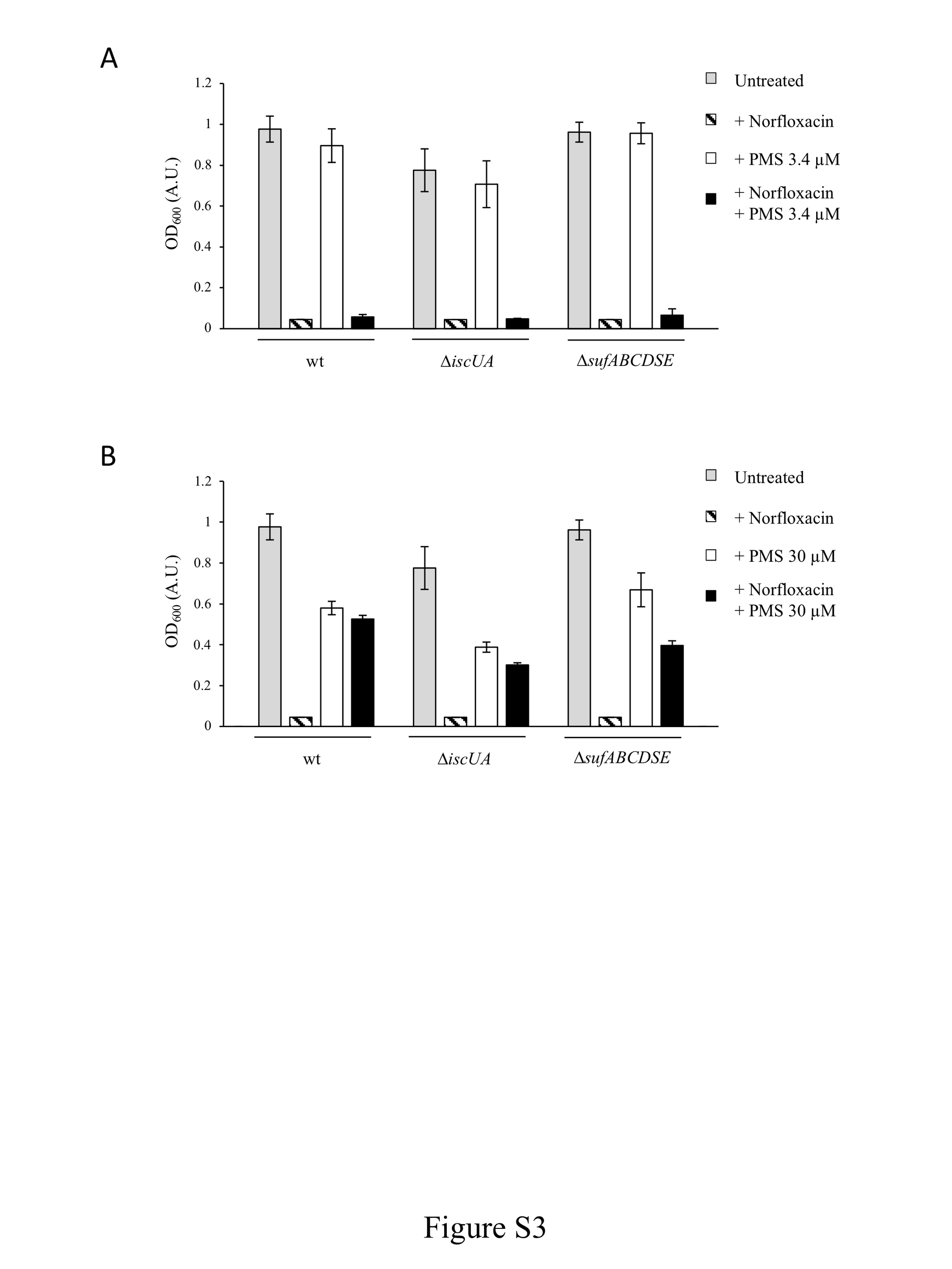
**

**S3 Fig. PMS-dependent induction of the P*soxS::lacZ* fusion and norfloxacin resistance**

The *E. coli* wt (BE1000), ∆*iscUA* (AG030), and ∆*sufABCDSE* (AG031) strains were grown to mid-log phase in LB and then diluted to inoculate 96-well microplate wells containing liquid LB medium (grey bars), LB medium supplemented with PMS (3.4 μM in panel A; 30 μM in panel B) (white bars), LB medium supplemented with norfloxacin (160 ng/mL) (hatched bars), and LB medium supplemented with both PMS and norfloxacin (160 ng/mL) (black bars). Cultures were incubated 18 hours at 37°C with shaking. Plates were read for OD_600_ in Tecan Infinite. The experiments were repeated at least three times. The means and standard deviations are shown.
