# Supplementary material for: Oxidative stress antagonizes fluoroquinolone drug sensitivity via the SoxR-SUF Fe-S cluster homeostatic axis
Source: PLoS Genet. 2020 Nov 2;16(11):e1009198. doi: 10.1371/journal.pgen.1009198 (PMC7671543; doi:10.1371/journal.pgen.1009198)
Supplement: S4 Fig — The E. coli strains carrying the chromosomal PsoxS::lacZ fusion, wt (BE1000) (black circles) and ΔnfuA (AG043) (white squares) were grown in LB until OD600 reached 0.2. At time zero, the cultures were treated with PMS (30 μM), and β-galactosidase activity was monitored and expressed as Miller units. The experiments were repeated at least three times. The means and standard deviations are shown. (DOCX) [file pgen.1009198.s006.docx]

**
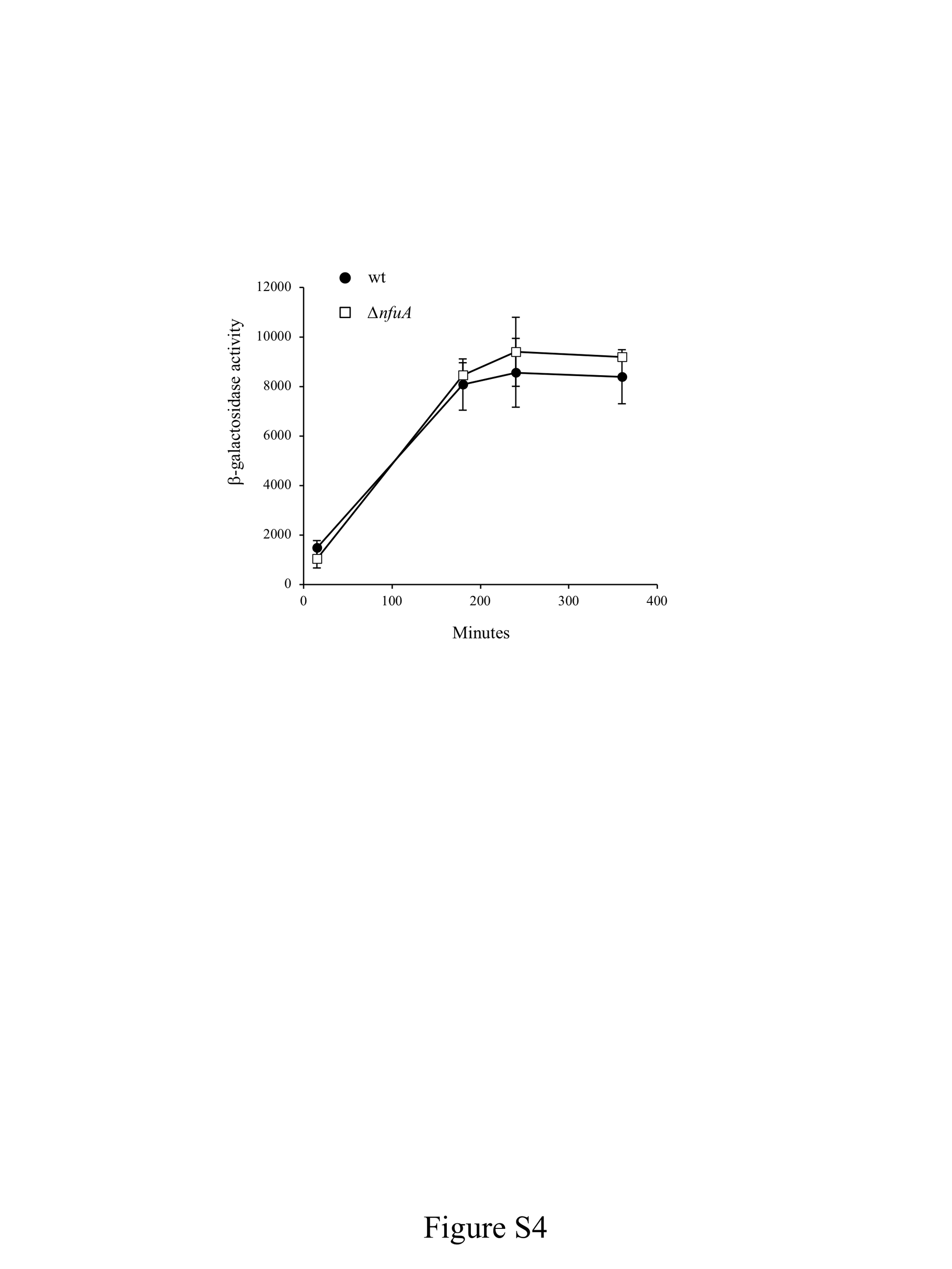
**

**S4 Fig. NfuA is dispensable for SoxR maturation during PMS stress.**

The *E. coli* strains carrying the chromosomal P*soxS::lacZ* fusion, wt (BE1000) (black circles) and ∆*nfuA* (AG043) (white squares) were grown in LB until OD_600_ reached 0.2. At time zero, the cultures were treated with PMS (30 µM), and β-galactosidase activity was monitored and expressed as Miller units. The experiments were repeated at least three times. The means and standard deviations are shown.
