# Supplementary material for: Oxidative stress antagonizes fluoroquinolone drug sensitivity via the SoxR-SUF Fe-S cluster homeostatic axis
Source: PLoS Genet. 2020 Nov 2;16(11):e1009198. doi: 10.1371/journal.pgen.1009198 (PMC7671543; doi:10.1371/journal.pgen.1009198)
Supplement: S5 Fig — (A-B) The E. coli strains carrying the chromosomal PsoxS::lacZ fusion, wt (BE1000) (black circles) and ParaBAD::erpA (AG048) (black crosses) (A-B), were grown overnight in LB and then inoculated (1/100) in fresh LB medium with glucose (0.2%) (A) or arabinose (0.2%) (B). The cultures were grown to an OD600 of 0.2, and were each split into two flasks, PMS (30 μM) was added (time zero) in one (solid line), and the other was left untreated (dotted line). All cultures were further incubated at 37°C with shaking. β-galactosidase activity was monitored and expressed as Miller units. (C) Growth of the E. coli wt strain (BE1000) containing the chromosomal PsoxS::lacZ fusion and carrying the plasmids pdCas9 together with the plasmid allowing erpA extinction, pRBS-erpA (white triangles), or the empty control vector, psgRNA (black circles). Cells were grown overnight in LB without anhydrotetracycline (aTc) and then inoculated (1/100) in fresh LB medium supplemented with aTc (2 μM). Growth was recorded by measuring OD600 following time. (D) The E. coli strain possessing the chromosomal PerpA::lacZ fusion (PM2040) and carrying the plasmid pdCAS9 together with the plasmid allowing erpA extinction, pRBS-erpA or the control vector, psgRNA. Cells were grown overnight in LB without anhydrotetracycline (aTc) and then inoculated (1/100) in fresh LB medium supplemented with aTc (2 μM). When cultures reached an OD600 of 0.2, aliquots were taken to assay β-galactosidase activity that is expressed in Miller units. (E-F) The E. coli wt strain possessing the chromosomal PsoxS::lacZ fusion (BE1000) and carrying the plasmid pdCas9 together with either the plasmid allowing erpA extinction, pRBS-erpA (white triangles), or the empty control vector, psgRNA (black circles) were grown overnight in LB and then inoculated (1/100) in fresh LB medium supplemented (E) or not (F) with aTc (2 μM). The cultures were grown to an OD600 of 0.2, and were each split into two flasks, PMS (30 μM) was added (time ze [file pgen.1009198.s007.docx]

**
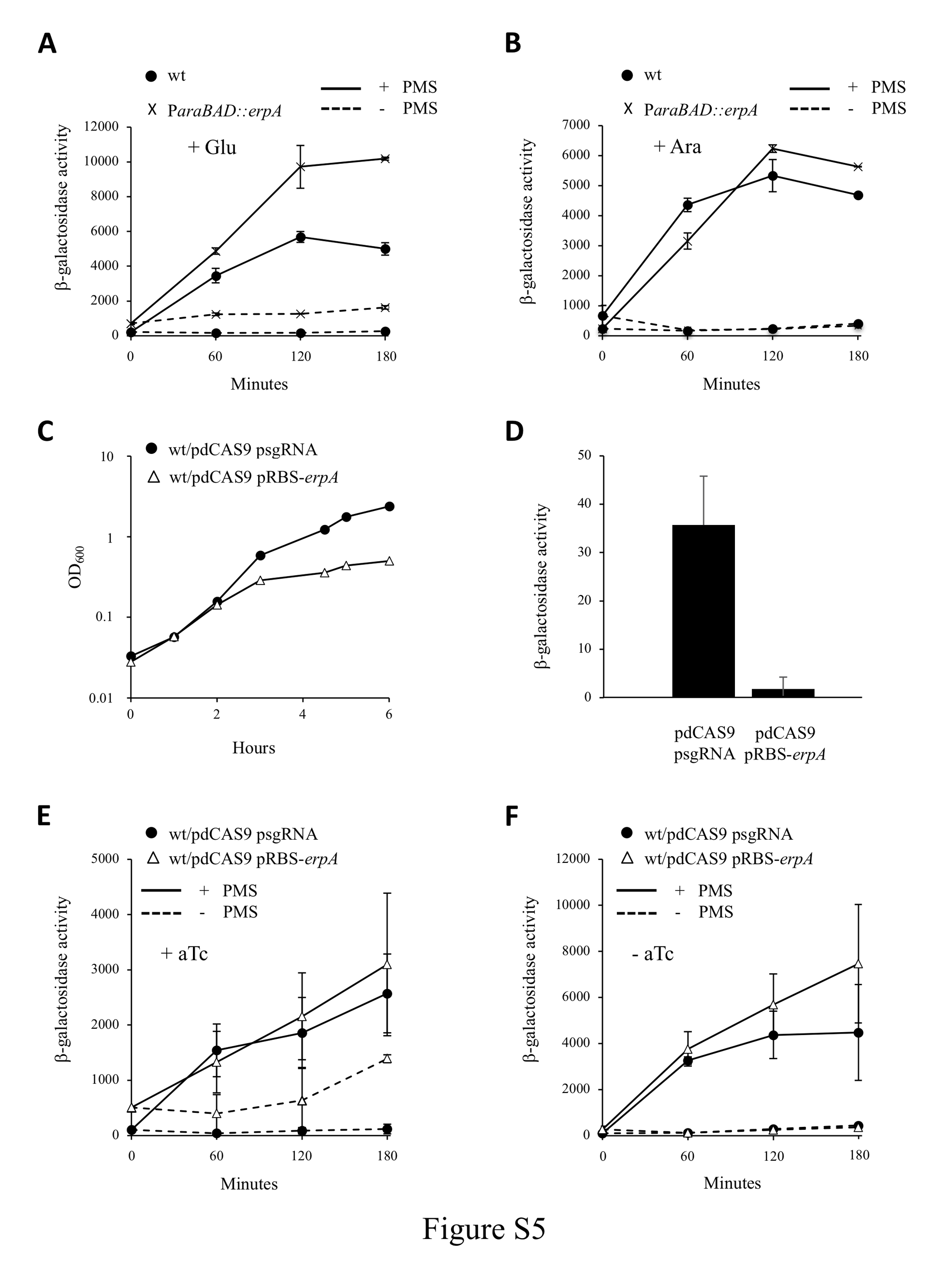
**

**S5 Fig. ErpA is not required for SoxR maturation.**

(A-B) The *E. coli* strains carrying the chromosomal P*soxS::lacZ* fusion, wt (BE1000) (black circles) and P*araBAD::erpA* (AG048) (black crosses) (A-B), were grown overnight in LB and then inoculated (1/100) in fresh LB medium with glucose (0.2 %) (A) or arabinose (0.2 %) (B). The cultures were grown to an OD_600_ of 0.2, and were each split into two flasks, PMS (30 µM) was added (time zero) in one (solid line), and the other was left untreated (dotted line). All cultures were further incubated at 37°C with shaking. β-galactosidase activity was monitored and expressed as Miller units. (C) Growth of the *E. coli* wt strain (BE1000) containing the chromosomal P*soxS::lacZ* fusion and carrying the plasmids pdCas9 together with the plasmid allowing *erpA* extinction, pRBS-*erpA* (white triangles)*,* or the empty control vector, psgRNA (black circles). Cells were grown overnight in LB without anhydrotetracycline (aTc) and then inoculated (1/100) in fresh LB medium supplemented with aTc (2 µM). Growth was recorded by measuring OD_600_ following time. (D) The *E. coli* strain possessing the chromosomal P*erpA::lacZ* fusion (PM2040) and carrying the plasmid pdCAS9 together with the plasmid allowing *erpA* extinction, pRBS-*erpA* or the control vector, psgRNA. Cells were grown overnight in LB without anhydrotetracycline (aTc) and then inoculated (1/100) in fresh LB medium supplemented with aTc (2 µM). When cultures reached an OD_600_ of 0.2, aliquots were taken to assay β-galactosidase activity that is expressed in Miller units. (E-F) The *E. coli* wt strain possessing the chromosomal P*soxS::lacZ* fusion (BE1000) and carrying the plasmid pdCas9 together with either the plasmid allowing *erpA* extinction, pRBS-*erpA* (white triangles)*,* or the empty control vector, psgRNA (black circles) were grown overnight in LB and then inoculated (1/100) in fresh LB medium supplemented (E) or not (F) with aTc (2 µM). The cultures were grown to an OD_600_ of 0.2, and were each split into two flasks, PMS (30 µM) was added (time zero) in one (solid line), and the other was left untreated (dotted line). All cultures were further incubated at 37°C with shaking and β-galactosidase activity was monitored and expressed as Miller units. All the experiments were repeated at least three times. The means and standard deviations are shown (A, B, D, E and F) and a representative experiment is presented in panel C.
